# Planar Cyclopenten‐4‐yl Cations: Highly Delocalized π Aromatics Stabilized by Hyperconjugation

**DOI:** 10.1002/anie.202009644

**Published:** 2020-08-25

**Authors:** Samuel Nees, Thomas Kupfer, Alexander Hofmann, Holger Braunschweig

**Affiliations:** ^1^ Institut für Anorganische Chemie Julius-Maximilians-Universität Würzburg Am Hubland 97074 Würzburg Germany; ^2^ Institute for Sustainable Chemistry & Catalysis with Boron Julius-Maximilians-Universität Würzburg Am Hubland 97074 Würzburg Germany

**Keywords:** ACID, carbocations, cyclopenten-4-yl cation, hyperconjugation, π aromaticity

## Abstract

Theoretical studies predicted the planar cyclopenten‐4‐yl cation to be a classical carbocation, and the highest‐energy isomer of C_5_H_7_^+^. Hence, its existence has not been verified experimentally so far. We were now able to isolate two stable derivatives of the cyclopenten‐4‐yl cation by reaction of bulky alanes Cp^R^AlBr_2_ with AlBr_3_. Elucidation of their (electronic) structures by X‐ray diffraction and quantum chemistry studies revealed planar geometries and strong hyperconjugation interactions primarily from the C−Al σ bonds to the empty p orbital of the cationic sp^2^ carbon center. A close inspection of the molecular orbitals (MOs) and of the anisotropy of current (induced) density (ACID), as well as the evaluation of various aromaticity descriptors indicated distinct aromaticity for these cyclopenten‐4‐yl derivatives, which strongly contrasts the classical description of this system. Here, strong delocalization of π electrons spanning the whole carbocycle has been verified, thus providing rare examples of π aromaticity involving saturated sp^3^ carbon atoms.

## Graphical abstract

Delocalized, not classical: Despite its structural relationship to the non‐classical 7‐norbornenyl system earlier studies predicted the cyclopenten‐4‐yl cation to be a localized, classical carbocation. We have now been able to capture two stable derivatives of this elusive species by making use of anchoring aluminyl substituents and established a highly delocalized π aromatic structure.

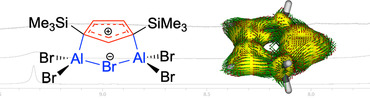

## Introduction

**Figure 1:**
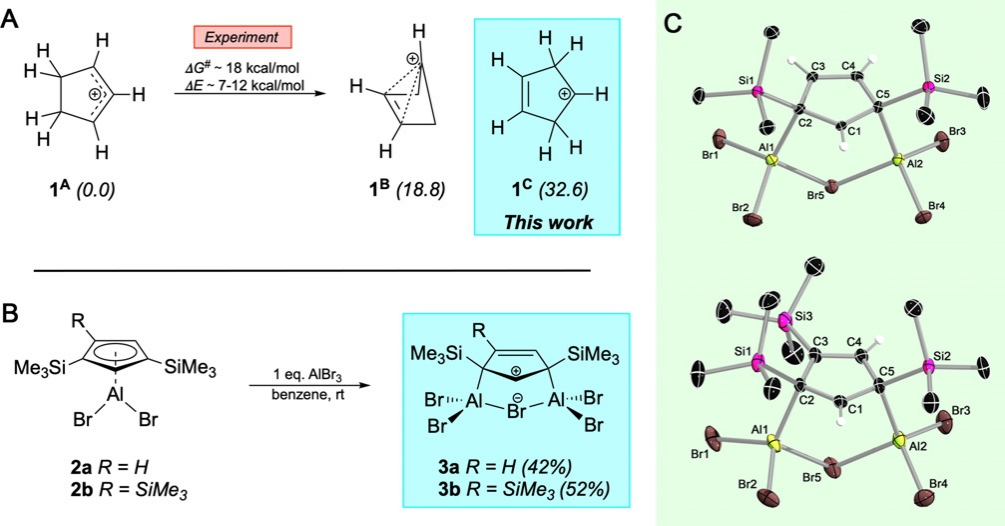
The cationic cyclopentenyl system. (**A**) Isomeric descriptions of the cyclopentenyl cation: allylic **1^A^**, bishomoaromatic **1^B^**, and planar **1^C^**. Reported relative energies (kcal mol^−1^) calculated at the MP3/6‐31G** level of theory are given in brackets.^12^ (**B**) Regioselective synthesis of cyclopenten‐4‐yl cations **3 a** and **3 b** by reaction of Cp‐substituted alanes **2 a** and **2 b** with one equivalent of AlBr_3_ in benzene. Isolated yields are given in brackets. (**C**) Molecular structures of **3 a** (top) and **3 b** (bottom) in the solid state. Only one molecule of the asymmetric unit of **3 a** is shown. Most of the hydrogen atoms have been omitted for clarity. Selected bond lengths (Å): **3 a** C1‐C2 1.435(4), C1‐C5 1.424(4), C2‐C3 1.477(4), C3‐C4 1.347(4), C4‐C5 1.479(4), Al1‐C2 2.031(3), Al2‐C5 2.038(3), Si1‐C2 1.937(3), Si2‐C5 1.943(3); **3 b** C1‐C2 1.427(4), C1‐C5 1.426(4), C2‐C3 1.492(4), C3‐C4 1.376(4), C4‐C5 1.469(4), Al1‐C2 2.033(3), Al2‐C5 2.043(3), Si1‐C2 1.955(3), Si2‐C5 1.939(3), Si3‐C3 1.894(3).

Recent interest on cyclopentenyl cations **1** stems from their intermediacy in important organic (Nazarov cyclization –formation of cyclopentenones)^1^, ^2^, ^3^, ^4^, ^5^ and industrial processes (methanol‐to‐hydrocarbon conversion).^6^, ^7^, ^8^, ^9^ Historically however, it was the presence of a C=C double bond in close proximity to a cationic sp^2^ carbon center, and hence the possibility of gaining “extra stabilization” by allylic or homoaromatic delocalization, that put this system into the focus of carbocation chemistry.^10^ From a theoretical point of view, three isomeric forms of the cyclopentenyl cation **1** appear plausible, that is, allylic **1^A^**, bishomoaromatic **1^B^**, and planar **1^C^** (Figure 1 A). Semi‐empirical and high‐level *ab initio* studies established allylic **1^A^** as the most stable isomer, being energetically favored by 18.8 kcal mol^−1^ over **1^B^** (MP3/6‐31G**).^11^, ^12^, ^13^ The bishomoaromatic structure **1^B^** itself is about 6–14 kcal mol^−1^ lower in energy (depending on the level of theory) than the classical planar structure **1^C^**, making the cyclopenten‐4‐yl cation (**1^C^**) the least favorable isomer. Early solvolysis studies are consistent with these findings, with allylic **1^A^** being the only observable isomer, notwithstanding the nature of the studied cyclopentene precursor.^14^, ^15^, ^16^, ^17^, ^18^ Thus, attempts to generate isomer **1^C^**, or its homoaromatic analog **1^B^**, by solvolysis of 4‐Br/OTs‐cyclopentene were unsuccessful. Instead allylic **1^A^** was immediately formed by 1,2‐hydride shift, indicating both the lability of **1^B^**/**1^C^** and the thermodynamic stability of allylic cation **1^A^**.^14^, ^16^, ^18^ This hydrogen scrambling process has been used to estimate the activation free energy Δ*G* ^#^ for the hydrogen migration step connecting stable **1^A^** and **1^B^**/**1^C^** by means of NMR line‐broadening/spin‐saturation transfer experiments (18.0±0.9 kcal mol^−1^)^17^ and low temperature ^2^H NMR spectroscopy (18.3±0.1 kcal mol^−1^).^18^ The energy difference Δ*E* between allylic cation **1^A^** and its high‐energy isomers **1^B^**/**1^C^** has been approximated to 6.7–8.6 kcal mol^−1^ and 12 kcal mol^−1^ by ion cyclotron measurements^11^ and mass spectrometric studies,^19^ respectively. Experimental studies also indicated that anchimeric assistance to solvolysis by the C=C double bond is absent in cyclopenten‐4‐yl systems (**1^B^**/**1^C^**), instead reduced rates of solvolysis as compared to their saturated analogs were noted.^12^, ^14^

This is in stark contrast to the results obtained for structurally related 7‐norbornenyl systems; the solvolysis rates are enhanced by a factor of up to 10^11^ over those of the saturated 7‐norbornyl analogs, affording carbocations of exceptional stability.^20^ These 7‐norbornenyl cations are dramatically stabilized by homoaromatic 3‐center‐2‐electron bonding,^10^ making them readily amenable for structural characterization even in the solid state.^21^, ^22^, ^23^, ^24^ Hence, solvolysis studies argue against any degree of bishomoaromatic delocalization in cyclopenten‐4‐yl cations (**1^B^**); thus a classical description as **1^C^** is usually preferred here. This view was further supported by low values for the calculated delocalization indices (DI=0.09) of the transannular C−C bonds in planar **1^C^**.^25^ It should be noted, however, that a different theoretical approach recently indicated significant bishomoaromatic delocalization in **1^C^**.^26^ Overall, it is not surprising that stable derivatives of the cyclopentenyl cation are only known for allylic isomer **1^A^**, including a small number of structurally characterized molecules.^27^, ^28^, ^29^, ^30^, ^31^, ^32^, ^33^ By contrast, derivatives of isomers **1^B^** and **1^C^** are still absent in the literature; only a dianionic boron analog of bishomoaromatic **1^B^** has been realized so far.^34^

Herein we report the experimental evaluation of the electronic structure of cyclopenten‐4‐yl cations. As part of our ongoing studies on aluminum molecules with bulky Cp ligands,^35^, ^36^, ^37^, ^38^, ^39^ we were now able to capture two stable derivatives of the elusive cyclopenten‐4‐yl cation **1^C^**. Their high stability and the regioselectivity of their formation are readily explained by strong hyperconjugation from C−Al and C−Si σ bonds into the empty p orbital of the cationic sp^2^ carbon. We have also examined the anisotropy of the current (induced) density (ACID)^40^, ^41^ and different aromaticity indicators, which revealed that the cyclopenten‐4‐yl cation should be considered a highly delocalized π aromatic system rather than a classical carbocation. Our results thus demonstrate that delocalization of π electrons can involve sp^3^ carbon centers via hyperconjugation effects, creating an alternative to homoaromaticity for electronic stabilization in cyclopenten‐4‐yl cations of the type **1^C^**.

## Results and Discussion

Recently, we prepared the aluminum reagent Cp^3t^AlBr_2_ featuring the bulky Cp^3t^ ligand (Cp^3t^=*η* ^5^‐1,2,4‐*t*Bu_3_‐C_5_H_2_), which allowed us to isolate a monomeric Cp^3t^Al(I) species for the first time.^37^ Exploration of its reactivity subsequently helped to unravel unknown reaction pathways,^37^, ^38^, ^39^ providing access to unprecedented molecules featuring for example Al−B multiple bonding.^39^ Hence, we set out to evaluate the impact of the substituents of the Cp ligand on the stability and reactivity of this system. Of particular interest for us was the replacement of the peripheral *t*Bu groups by Me_3_Si groups to assess the divergent electronic effects of silicon *vs*. carbon, while at the same time keeping the sterics rather unaltered. Thus, we attempted the syntheses of Cp^2Si^AlBr_2_ (**2 a**; Cp^2Si^=*η* ^5^‐1,3‐(Me_3_Si)_2_‐C_5_H_3_) and Cp^3Si^AlBr_2_ (**2 b**; Cp^3Si^=*η* ^5^‐1,2,4‐(Me_3_Si)_3_‐C_5_H_2_) by reacting two equivalents of AlBr_3_ with either (Cp^2Si^)_2_Mg or (Cp^3Si^)_2_Mg in benzene solutions at room temperature. Due to the high solubility of the magnesium salts in aromatic solvents, the reactions proceeded smoothly and quantitatively within 2–4 hours, and molecules **2 a** (64 %) and **2 b** (83 %) were isolated as off‐white solids. The identity of **2 a** and **2 b** was readily verified by NMR spectroscopy, elemental analysis, and in the case of **2 b**, X‐ray diffraction (Figure S16). Characteristic solution NMR parameters of **2 a** and **2 b** include a single ^27^Al NMR resonance (**2 a** −32 ppm; **2 b** −39 ppm), and ^1^H NMR resonances for the aromatic protons (**2 a** 6.75, 7.05 ppm; **2 b** 7.42 ppm) and the peripheral Me_3_Si protons (**2 a** 0.23 ppm; **2 b** 0.27, 0.38 ppm) with chemical shifts similar to those of Cp^3t^AlBr_2_.^37^

When the reactions were carried out with a larger excess of AlBr_3_ (approx. 4 equiv.), however, we were surprised that a second equivalent of AlBr_3_ was consumed along with a slight darkening of the pale‐yellow benzene solutions. ^27^Al NMR spectroscopy suggested gradual conversion of **2 a** and **2 b** into new aluminum‐containing species with broad ^27^Al NMR signals at 102 ppm (**3 a**, **3 b**). The large low‐field shifts of these signals are indicative of substantial changes around the aluminum centers. Full conversion of **2 a** and **2 b** was achieved at room temperature within approximately 12 hours. In both cases, we were able to separate colorless cyclopenten‐4‐yl cations **3 a** (27 %) and **3 b** (30 %) in moderate yields from the reaction mixtures. We note that the synthesis of **3 a** and **3 b** can also be accomplished by reacting isolated **2 a** and **2 b** with one equivalent of AlBr_3_, which notably simplifies purification of the cyclopenten‐4‐yl cations, and allows for their large‐scale isolation in yields of 42 % (**3 a**) and 52 % (**3 b**) (Figure 1 B). For convenience, this method is the preferred one. The formation of cationic cyclopenten‐4‐yl systems is clearly evident from the ^1^H and ^13^C NMR spectra of **3 a** in solution, which show signal patterns and chemical shifts consistent with such a structural motif. Accordingly, the carbocationic sp^2^ center is significantly deshielded, giving rise to low‐field ^1^H (9.00 ppm) and ^13^C NMR signals (192.88 ppm) for the C^+^‐H moiety. In addition, the NMR spectra show signals at 7.06 ppm (^1^H) and 144.79 ppm (^13^C) for the alkenyl C−H moieties, while the quaternary sp^3^ carbons entail a ^13^C NMR resonance at 106.68 ppm.

**Figure 2:**
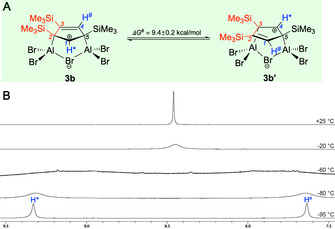
Fluxional behavior of **3 b**. (**A**) Rapid degenerate 1,2‐shift observed for **3 b**. (**B**) VT NMR spectra of **3 b** recorded in [D_8_]toluene, freezing the process at *T*_C_=−57 °C.

By contrast, compound **3 b** shows fluxional behavior in solution (Figure 2); the carbocationic center switches between the two C−H fragments by means of reversible 1,2‐shifts of one bridging aluminum center between two neighboring Me_3_Si‐C moieties. As a consequence, only a single ^1^H NMR resonance is observed for the C−H groups of the cyclopenten‐4‐yl system in **3 b** at room temperature (C_*1*_‐H*, C_*4*_‐H^#^), shifted somewhat to higher field (8.50 ppm). Moreover, the ^13^C NMR spectrum of **3 b** shows a slightly high‐field shifted averaged signal for the sp^2^ carbon centers C_*1*_ and C_*4*_ (176.82 ppm), an averaged signal for the sp^2^/sp^3^ centers C_*2*_ and C_*3*_ (137.77 ppm), and a resonance for the quaternary sp^3^ carbon C_*5*_ (105.29 ppm). This process can be frozen at low temperatures featuring a coalescence temperature *T*_C_ of about −57 °C (Figures 2 B, S13). At temperatures as low as −95 °C, sharp and distinct signals become evident in the ^1^H and ^13^C NMR spectrum of **3 b** with the expected low‐field signals for the C_*1*_^+^‐H* moiety (^1^H 9.29 ppm; ^13^C 196.39 ppm), as well as separate resonances for the alkenyl (^1^H 7.60 ppm; ^13^C 154.86 (C_*4*_), 159.36 (C_*3*_) ppm) and sp^3^ carbon entities (^13^C 103.55 (C_*5*_), 110.44 (C_*2*_) ppm). Assuming a coalescence temperature *T*_C_=−57 °C, a rate constant *k*_C_=1505 s^−1^, and a free enthalpy of activation Δ*G* ^#^_C_=9.4±0.2 kcal mol^−1^ can be approximated for this process. These findings are supported by quantum chemistry, which provided a similar activation barrier for the interconversion **3 b→3 b′** (Δ*G* ^#^_**3 b**_ **→_3 b′_**=8.9 kcal mol^−1^), as well as small differences in thermal free energy between both isomers (Δ*E*_298_=0.7 kcal mol^−1^), thus verifying the degenerate nature of this rearrangement. Nevertheless, the regioselectivity of cyclopenten‐4‐yl cation formation is high, and only regioisomers with the carbocationic center trapped in‐between two quaternary Si/Al‐substituted carbon atoms, that is, the 3,5 (**3 a**) and 1,3,5 (**3 b**) isomers are observable in the NMR spectra of the reaction mixtures and isolated molecules.

The results of X‐ray diffraction studies on single crystals of **3 a** and **3 b** are consistent with NMR spectroscopy in solution, and therefore further justify their description as derivatives of the cyclopenten‐4‐yl cation **1^C^** (Figure 1 C). The key feature of the molecular structures of **3 a** and **3 b** is their central C_5_ carbocycle, which is essentially planar for both molecules (torsion angles: **3 a** −1.1(3)° to 1.2(3)°; **3 b** −1.5(3)° to 1.5(3)°). The C_5_ rings contain a cationic sp^2^ carbon center C1 (**3 a** *Σ*=360.0°; **3 b** *Σ*=360.0°), which is directly linked to two quaternary sp^3^ carbon atoms C2 and C5. The C1‐C2 and C1‐C5 distances are in the range of 1.424(4)–1.435(4) Å, thus they are intermediate of typical C−C single (1.53 Å) and C=C double bonds (1.32 Å),^42^ which implies the presence of strong hyperconjugation from the adjacent C−Si and C−Al σ bonds. The stabilization of positive charge by groups in β position is a well documented phenomenon, particularly for silicon and its higher homologs (*cf. β effect of silicon*).^43^, ^44^, ^45^, ^46^, ^47^, ^48^, ^49^, ^50^ Similar bond lengths were reported for a couple of structurally characterized carbocations, such as Me_3_C^+^, MeC_5_H_8_^+^, or Me_2_CPh^+^.^51^, ^52^ Short C3−C4 bonds (**3 a** 1.347(4) Å; **3 b** 1.376(4) Å) are reminiscent of isolated C=C double bonds, and their distances approach values found in cycloalkene derivatives (1.33 Å).^42^ The alkenyl and carbocationic fragments are connected via C2−C3 and C4−C5 with bond lengths between 1.469(4)–1.492(4) Å, thus suggesting considerable C−C single bond character. Hence, all structural criteria usually associated with cyclopenten‐4‐yl cations are also present in **3 a** and **3 b**. Their molecular structure is completed by a bridging Br_2_Al‐Br‐AlBr_2_ loop attached to the sp^3^ carbons C2 and C5 via Al1 and Al5, respectively, with Al1‐C2 and Al2‐C5 distances (2.031(3)–2.043(3) Å) that fall within the range of reported covalent Al−C bonds (1.98 2.09 Å).^38^, ^53^, ^54^ It is this Al−C bond covalency that enables the Br_2_Al‐Br‐AlBr_2_ loop to (formally) act as counter anion, eventually affording stable, neutral bicyclic species **3 a** and **3 b**. As a side note, it should be mentioned here that (*i*) the reactions of **2 a**/**2 b** with one equivalent of AlCl_3_, or (*ii*) those of Cp^R^AlCl_2_ (Cp^R^=Cp^2Si^, Cp^3Si^) with AlCl_3_ also ended in the generation of related (mixed) cyclopenten‐4‐yl systems, as implied by *in situ* ^1^H and ^27^Al NMR studies. However, reactions proceeded less selectively, and we were thus far not able to isolate any of these molecules.

**Figure 3:**
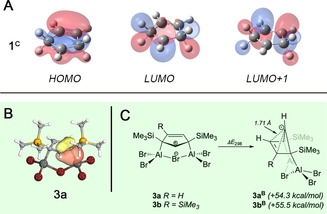
Theoretical studies on **3 a** and **3 b**: evaluation of the electronic structure, the regioselectivity of their formation, and their stability. (**A**) Frontier molecular orbitals (MOs) of **1^C^**: HOMO, LUMO, and LUMO+1 (isosurface plots at 0.04 a.u.). (**B**) Representative example for the intrinsic bonding orbitals (IBOs) of **3 a** illustrating hyperconjugation. (**C**) Calculated differences in thermal free energies (Δ*E*_298_) for the conversion of planar (**1^C^**‐type) to bishomoaromatic, bridged (**1^B^**‐type) structures of **3 a** and **3 b**.

We next investigated the electronic structures of these uncommon cyclopenten‐4‐yl derivatives by quantum chemical calculations aiming at understanding the reason for their regioselective formation and their remarkable stability. To this end, **3 a** and **3 b** were studied by density functional theory (DFT) at the SMD‐M06L/Def2‐TZVP//M06L/Def2‐SVP level of theory. Calculated structural and spectroscopic parameters of **3 a** and **3 b** agree very well with experimentally determined values (Supporting Information). First, we inspected their frontier molecular orbitals (MOs), and compared them to those calculated for the parent C_5_H_7_^+^ cation (**1^C^**) at the same level of theory, in order to ascertain the presence of similar electronics, eventually justifying a description of **3 a** and **3 b** as derivatives of the cyclopenten‐4‐yl cation **1^C^** (Figures S45–S52). This is indeed the case, and relevant MOs are of very similar shape for all three species. The highest occupied molecular orbital (HOMO) is of π symmetry with major contributions from the π orbital of the C=C double bond and the empty p orbital at carbocationic C1 (including hyperconjugation effects). The lowest unoccupied molecular orbital (LUMO) represents the antibonding part of the hyperconjugation interactions between the C−Al/H and C−Si/H σ bonds of the quaternary carbon atoms and the adjacent empty p orbital at C1, while LUMO+1 illustrates their antibonding interactions with the π* orbital of the alkenyl moiety (**1^C^**: Figure 3 A; **3 a**, **3 b**: Figures S47–S52). We note that a full analysis of the MOs of **3 a**, **3 b**, and **1^C^**, to our surprise, indicated a large extent of delocalization of π electrons (*cf*. HOMOs) spanning the whole C_5_ carbocycle, which strongly conflicts with the earlier description of **1^C^** as classical carbocation.

The nature of hyperconjugation effects in **3 a** and **3 b** was assessed by natural bonding orbital (NBO) analysis and by examination of the intrinsic bonding orbitals (IBOs). Second order perturbation theory revealed highly stabilizing hyperconjugation interactions involving combinations of the p orbital lone vacancy at C1 (acceptor NBO) and donor NBOs reminiscent of C−Al *σ* (**3 a**: −50.7, −54.5 kcal mol^−1^; **3 b**: −52.1, −56.1 kcal mol^−1^) and C−Si σ bonding (**3 a**: −16.7, −17.5 kcal mol^−1^; **3 b**: −17.8, −17.8 kcal mol^−1^), which readily account for the high stability of the planar cyclopenten‐4‐yl structures of **3 a** and **3 b**.^43^, ^44^, ^45^, ^46^, ^47^, ^48^, ^49^, ^50^ We are aware that these energetic contributions appear remarkably high, particularly as compared to previous studies on hyperconjugation effects,^55^ which tentatively is related to the delocalized nature of **3 a** and **3 b**; thus, the energies rather define the upper boundary of stabilizing hyperconjugation interactions. The intrinsic bonding analysis of **3 a** and **3 b** gave similar results, and sets of IBOs were found for both molecules that visualize hyperconjugation from C−Si and C−Al σ bonds to the vacant p orbital at the carbocationic center (Figures 3 B, S53 and S54). We note that the shape of these IBOs is very similar to that of the natural localized molecular orbitals (NLMOs) relevant to hyperconjugation as determined by NBO analysis. Such stabilizing interactions are naturally absent in the respective hypothetical bishomoaromatic isomers **3 a^B^** and **3 b^B^** (Figure 3 C) due to geometry constraints imposed by the highly bent structure. These molecules were optimized starting with key structural parameters derived from earlier calculations (C1−C3/C4 1.82 Å),^12^ and eventually converged to energy minima structures with transannular C1−C3/C4 distances of ca. 1.71 Å. Accordingly, thermal free energies *E*_298_ of bishomoaromatic **3 a^B^** and **3 b^B^** are considerably higher than those of planar **3 a** and **3 b** (**3 a**: +54.3 kcal mol^−1^; **3 b**: +55.5 kcal mol^−1^), which fundamentally contrasts with the findings obtained for parent C_5_H_7_^+^. Thus, our calculations suggest strong Al−C hyperconjugation as one important factor that allow for the isolation of molecules **3 a** and **3 b** with a planar, cationic cyclopenten‐4‐yl moiety. Natural resonance theory (NRT) treatment of **3 a** and **3 b** considering monocyclic **3 a**/**b** ^LS2^, **3 a**/**b** ^LS3^, and charge‐separated **3 a**/**b** ^LS4^ as other plausible Lewis formulas showed that delocalized Lewis structures **3 a** (87.8 %) and **3 b** (85.2 %) are by far the most appropriate descriptions (Figure S67).

**Figure 4:**
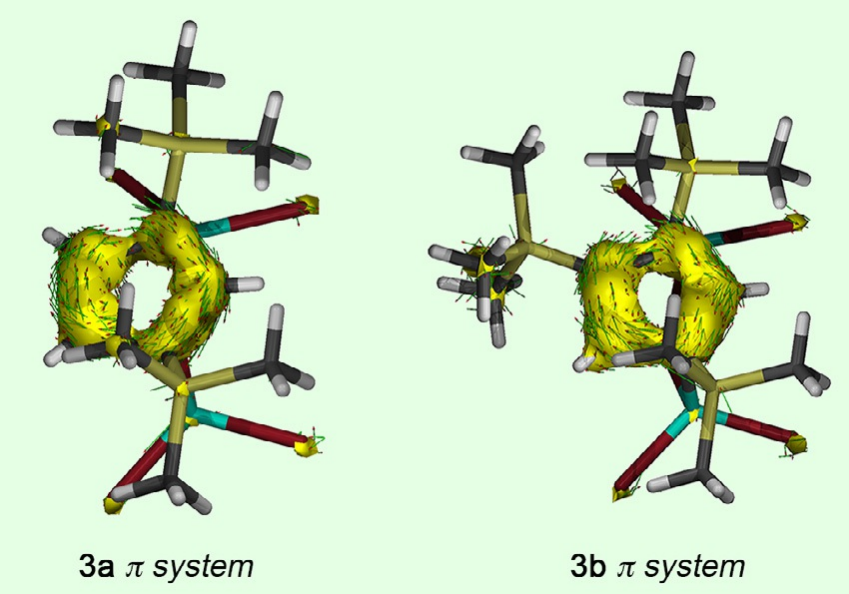
ACID isosurfaces of the π system of **3 a** and **3 b** at 0.05 a.u. Current density vectors are plotted onto the ACID isosurfaces to indicate diatropic (aromatic) ring currents. The magnetic field is orthogonal with respect to the molecular ring plane of the C_5_ carbocycle (clockwise currents are diatropic).

Subsequently, we tried to evaluate the nature and degree of electron delocalization in **3 a** and **3 b**, as indicated by their MO analysis. Initially, ring currents associated with this phenomenon were analyzed by the anisotropy of current (induced) density method,^40^, ^41^ wherein the applied magnetic field was oriented orthogonal to the molecular plane of the central C_5_ carbocycle pointing upward (Figures 4, and S61–S66). Hence, the current density vectors plotted on the ACID isosurfaces of **3 a**, **3 b**, and **1^C^** indicate strong diatropic (clockwise) ring currents for all three species, which substantiates the presence of significant delocalization (aromaticity) in these cyclopenten‐4‐yl systems. The critical isosurface values (CIV) were determined to be 0.060 (**3 a**), 0.061 (**3 b**), and 0.071 (**1^C^**), thus being only slightly lower than the value of benzene (0.074).^41^ To determine the origin of delocalization, we reconsidered the MOs in detail, aiming at separation of π contributions to the total ACID isosurfaces. For all three species we were able to clearly identify three π‐type MOs formed in large part by p_z_ orbitals of the planar C_5_ ring. Their ACID isosurfaces imply strong diatropic ring currents for the π system above and underneath the ring plane. Consequently, cyclopenten‐4‐yl cations **3 a**, **3 b**, and **1^C^** are to be considered π aromatics with the π electrons being highly delocalized over the complete C_5_ carbocycle involving both sp^3^ carbon centers. By contrast, we were not able to unambiguously identify all σ‐type MOs of the ring C−C σ bonds due to distinct interference of substituent orbitals. For **1^C^** however, consideration of all σ‐type orbitals for its ACID analysis revealed a weak paratropic (antiaromatic) ring current for the σ framework in the molecular C_5_ plane (Figures S55–S57); similar findings have been reported for the prototype system benzene.^41^ We note that cationic **3 a^+^** (0.054) and **3 b^+^** (0.056), which lack the bridging bromide Br5, provided similar CIVs for their total ACID isosurfaces, which makes the impact of the Br_2_Al‐Br‐AlBr_2_ loop on the delocalization of the systems negligible. Even though the concept of π aromaticity is well‐known for centuries, we did not expect it to be present for cyclopenten‐4‐yl cations, keeping in mind its assumed classical carbocation nature.

The π aromaticity encountered in **1^C^**, **3 a**/**3 b**, and **3 a^+^**/**3 b^+^** can readily be quantified and put into relationship to other classical (non)aromatic molecules by close analysis of suitable aromaticity descriptors, either based on electronic, magnetic, or structural criteria (Table 1). Particularly indicators based on electronic criteria such as the aromatic fluctuation index (FLU),^56^ or the multicenter indices *mc*‐DI (multicenter delocalization index),^57^ *I*_ring_,^58^ and MCI^59^ have recently emerged as powerful tools for accurate aromaticity estimates. As illustrated by the numbers in Table 1, all cylcopenten‐4‐yl cations studied here are clearly aromatic systems with the different electronic descriptors showing the same trends. Accordingly, parent **1^C^** exhibits the lowest degree of aromaticity in this series, while values of **3 a**/**3 b** and **3 a^+^**/**3 b^+^** strongly resemble each other. In general, their aromaticity is less pronounced (approx. 33–50 %) as in prototypical aromatics such as C_6_H_6_, NC_5_H_5_, and BC_5_H_5_, which is, however, not surprising given the presence of quaternary sp^3^ carbon centers within the planar C_5_ ring of **3 a**/**3 b** and **3 a^+^**/**3 b^+^**. Nonetheless, all cyclopenten‐4yl cations are considerably more aromatic than borazine B_3_N_3_H_6_ and (nonaromatic) cyclohexane.

**Table 1:** Aromaticity descriptors used to study electron delocalization in **1^C^**, **3 a**, **3 b**, **3 a^+^**, **3 b^+^**, and selected reference molecules (SMD‐M06L/def2‐TZVP).

	NICS(1)_zz_^[a]^	NICS(0)_πzz_^[a]^	NICS(0)_π_^[a]^	NICS(0)_iso_^[a]^	HOMA	*mc*‐DI^[b]^	FLU^[b]^	MCI^[b]^	*I*_ring_^[b]^
**1^C^**	−11.2	−20.9	−12.0	−2.3	−0.042	3.1	15.4	5.9	7.5
**3 a**	−23.1	−25.0	−14.4	−5.7	0.48	5.6	14.4	33.2	24.9
**3 b**	−22.7	−21.7	−11.0	−5.0	0.43	5.4	14.4	31.9	24.4
**3 a^+^**	−20.4	−21.2	−10.4	−5.1	0.49	6.2	14.6	34.5	26.2
**3 b^+^**	−19.7	−22.3	−10.9	−4.4	0.44	5.9	15.0	33.2	25.4
C_6_H_6_	−28.9	−36.7	−24.5	−8.3	1	20.5	0	72.1	48.1
NC_5_H_6_	−28.4	−36.6	−25.0	−7.1	0.97	19.8	4.1	66.0	44.7
BC_5_H_6_	−25.4	−33.0	−24.6	−11.9	0.99	17.4	–^[c]^	43.7	29.7
B_3_N_3_H_6_	−6.3	−17.1	−14.3	−1.7	0.95	5.5	–^[c]^	2.0	1.6
C_6_H_12_	–	–	–	1.9	−2.47	0.1	91.8	0.3	0.3

[a] Given in ppm. [b] Values multiplied by 1000. With the exception of FLU, the degree of aromaticity rises with increasing indices. [c] No parameters implemented for B−C and B−N bonds.

Similar results are obtained for aromaticity indicators based on magnetic criteria (Table 1), which also implied strong aromatic delocalization of electron density. For example, the nucleus‐independent chemical shift 1 Å above and orthogonal to the ring plane (NICS(1)_zz_) has frequently been used as reliable aromaticity index.^60^ Highly negative NICS(1)_zz_ values were calculated for **3 a**/**3 b** and **3 a^+^**/**3 b^+^** (−19.7 to −23.1 ppm), which approach values found for the classical aromatic reference molecules C_6_H_6_ (−28.9 ppm), NC_5_H_5_ (−28.4 ppm), and BC_5_H_5_ (−25.4 ppm), and clearly outmatch that of borazine (−6.3 ppm). We note that, even if NICS(1)_zz_ of parent **1^C^** (−11.2 ppm) is somewhat smaller than those of isolated **3 a** and **3 b**, **1^C^** still shows significant aromaticity.

This delocalization is not present for related carbocyclic model systems C_5_H_7_^−^ (−1.6/−3.4 ppm), C_5_H_9_^+^ (−3.5/−1.5 ppm), and neutral C_5_H_8_ (−2.1/−3.1 ppm) in their non‐planar, energy minimum configurations, as well as in their forced planar structures (given in italics; Supporting Information). Thus, only the combination of a C=C double bond with a neighboring empty p orbital qualifies the C_5_ system for aromatic delocalization. The origin of aromatic delocalization becomes evident when applying dissected NICS methods based on canonical MOs (CMO‐NICS) with NICS(0)_πzz_ currently being the most accurate index for the description of planar π rings.^60^ The highly negative NICS(0)_πzz_ values of all cyclopenten‐4‐yl cations (−20.9 to −25.0 ppm) clearly identify these molecules as strong π aromatics (*cf*. C_6_H_6_, NC_5_H_5_, BC_5_H_5_ −33.0 to −36.6 ppm; B_3_N_3_H_6_ −17.1 ppm). For completion, we included the isotropic CMO‐dissected NICS(0)_π_ index in Table 1, which essentially revealed the same trend. When comparing the NICS(0)_πzz_ to the values determined for the original isotropic NICS(0)_iso_ index (which includes local σ contributions),^61^ it becomes clear that the σ framework of **1^C^**, **3 a**/**3 b**, and **3 a^+^**/**3 b^+^** possesses antiaromatic character, which, however, is over‐compensated by strong contributions of the aromatic π system (*cf*. ACID analysis).

By contrast to electronic and magnetic indicators, aromaticity descriptors based on geometrical criteria (bond‐length equalization) such as the most commonly applied harmonic oscillator model of aromaticity (HOMA)^62^ were found to be not reliable for our cyclopenten‐4‐yl molecules. As shown in Table 1, HOMA values calculated for **3 a**/**3 b** and **3 a^+^**/**3 b^+^** (0.43–0.49) differ significantly from those of classical aromatics (0.95–1), which is again related to the hybridization differences of the ring carbon atoms (sp^2^ *vs*. sp^3^).

**Figure 5:**
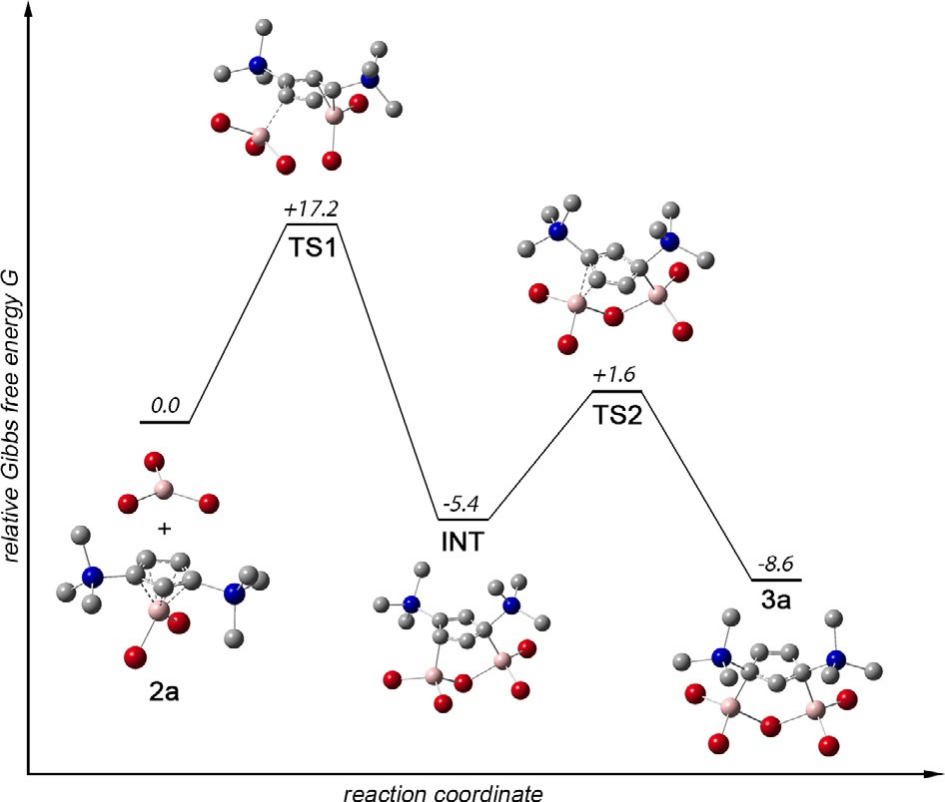
Plausible mechanism for the formation of cyclopenten‐4‐yl cation **3 a** by reaction of **2 a** with AlBr_3_. Relative Gibbs free energies *G* are given in kcal mol^−1^.

The mechanism of cyclopenten‐4yl cation formation was modelled by quantum chemistry exemplarily for **3 a**, and a plausible (two‐step) mechanism is illustrated in Figure 5. For simplicity, we presumed partial dissociation of Al_2_Br_6_ into monomeric AlBr_3_ in benzene solution. Accordingly, reaction of **2 a** and AlBr_3_ proceeds via concerted transition state **TS1** to initially afford the 1,3‐regioisomer **INT** as intermediate. This process is both exergonic by −5.4 kcal mol^−1^ (Gibbs free energy Δ*G*_1_), and exothermic by −18.0 kcal mol^−1^ (thermal free energy Δ*E*_1_). The formation of **TS1** involves hapticity change from *η* ^5^ to *η* ^2^ in **2 a**, and concomitant backside approach of AlBr_3_ to the less hindered carbon site C4. The activation barrier Δ*G* ^#^_1_ was calculated to be +17.2 kcal mol^−1^, thus it is readily accessible under the reaction conditions. The second step of this sequence, that is, rearrangement of **INT** to the experimentally observed 3,5 regioisomer **3 a** by means of 1,2 shift of the aluminum center Al2, is also energetically favorable (Δ*G*_2_=−3.2 kcal mol^−1^; Δ*E*_2_=−3.3 kcal mol^−1^). **INT** and **3 a** are connected by low‐barrier transition state **TS2** (Δ*G* ^#^_2_=+7.0 kcal mol^−1^), in which the aluminum center Al2 features a *η* ^2^‐type interaction to the C4‐C5 linkage. Overall, formation of **3 a** by reaction of **2 a** with AlBr_3_ is exergonic by Δ*G*=−8.6 kcal mol^−1^, and exothermic by Δ*E*=−21.3 kcal mol^−1^. We note that thermodynamics for the reaction of **2 b** with AlBr_3_ to afford **3 b** are similar (Δ*G*=−8.1 kcal mol^−1^; Δ*E*=−20.6 kcal mol^−1^), thus we expect a comparable mechanism to be valid for **3 b**, as well.

## Conclusion

Taken together, we succeeded in the isolation of two stable derivatives, **3 a** and **3 b**, of the cyclopenten‐4‐yl cation **1^C^**, a species that has eluded observation so far. While earlier calculations have described planar **1^C^** as a classical carbocation, and the most unfavorable isomer within the cyclopentenyl cation series, the presence of two aluminyl substituents provides sufficient stabilization via hyperconjugation interactions to allow for the regioselective generation and isolation of **3 a** and **3 b**. Inspection of the solid‐state structures (X‐ray), and elucidation of the electronics (DFT) served to verify their identity as cyclopenten‐4‐yl cation derivatives. By contrast to earlier findings, **3 a** and **3 b** are no classical carbocations, but rather highly delocalized systems featuring distinct π aromaticity involving the whole C_5_ carbocycle, that is, spanning the two quaternary sp^3^ centers.

## Conflict of interest

The authors declare no conflict of interest.

## Supporting information

As a service to our authors and readers, this journal provides supporting information supplied by the authors. Such materials are peer reviewed and may be re‐organized for online delivery, but are not copy‐edited or typeset. Technical support issues arising from supporting information (other than missing files) should be addressed to the authors.

Supplementary
