# Supplementary material for: Planar Cyclopenten‐4‐yl Cations: Highly Delocalized π Aromatics Stabilized by Hyperconjugation
Source: Angew Chem Int Ed Engl. 2020 Aug 25;59(42):18809–15. doi: 10.1002/anie.202009644 (PMC7589364; doi:10.1002/anie.202009644)
Supplement: Supplementary file 1 — Supplementary [file ANIE-59-18809-s001.pdf]

Angewandte Chemie
Eine Zeitschrift der Gesellschaft Deutscher Chemiker

Supporting Information

Planar Cyclopenten-4-yl Cations: Highly Delocalized π Aromatics Stabilized by Hyperconjugation

*Samuel Nees, Thomas Kupfer, Alexander Hofmann, and Holger Braunschweig**

anie_202009644_sm_miscellaneous_information.pdf

S1 Synthetic details and characterization of compounds

General experimental considerations: All manipulations were performed either under an atmosphere of dry argon, or in vacuo using standard Schlenk line or glovebox techniques. Deuterated solvents were dried over molecular sieves and degassed by three freeze-pump-thaw cycles prior to use. All other solvents were distilled and degassed from appropriate drying agents. Solvents (both deuterated and non-deuterated) were stored under argon over activated 4 Å molecular sieves. All glassware was oven-dried prior to use. $(Cp^{2Si})_2Mg$,[1] and $(Cp^{3Si})_2Mg$,[2] were prepared according to literature methods. $AlBr_3$ was purified by sublimation prior to use. NMR spectra were acquired on a Bruker Avance 400 NMR spectrometer (^{1}H: 400.1 MHz, ^{13}C: 101 MHz, ^{27}Al: 104 MHz). Chemical shifts (δ) are given in ppm and internally referenced to the carbon nuclei ($^{13}C\{^{1}H\}$) or residual protons (^{1}H) of the solvent. NMR spectra were referenced to $SiMe_4$ (^{1}H, ^{13}C), and $Al(NO_3)_3$ (^{27}Al) as external standards. All spectra were acquired at 298 K, unless stated otherwise. Resonances are given as singlet (s), doublet (d), or triplet (t). Microanalyses (C, H, N) were performed on an Elementar vario MICRO cube elemental analyser.

Synthesis and characterization of $Cp^{2Si}AlBr_2$ 2a

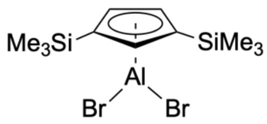

$(Cp^{2Si})_2Mg$ (250 mg, 564 μmol) was dissolved in benzene (25 mL), and $AlBr_3$ (301 mg, 1.13 mmol) was added. The solution turned yellow in colour, and a white precipitate formed immediately. After four hours at room temperature, the solution was filtered, and all volatiles were removed in vacuo. The residue was washed with cold pentane (20 mL) to afford **2a** as an off-white powder (287 mg, 724 μmol, 64%). All attempts to obtain crystals suitable for X-Ray diffraction failed.

^{1}H NMR (400.1 MHz, C_6D_6): δ = 0.23 [s, 18H, $(\mathit{Me}_3Si)_2C_5H_3$], 6.76 [d, 2H, $(Me_3Si)_2C_5\mathit{H}_3$, $^3J_{HH}$ = 2.0 Hz], 7.05 [t, 1H, $(Me_3Si)_2C_5\mathit{H}_3$, $^3J_{HH}$ = 1.9 Hz].

^{13}C NMR (101 MHz, C_6D_6): δ = –0.77 [s, $(\mathit{Me}_3Si)_2C_5H_3$], 120.80, 125.06, 131.89 [s, $(Me_3Si)_2\mathit{C}_5H_3$].

^{27}Al NMR (104 MHz, C_6D_6): δ = –32.

Elemental analysis calculated for $C_{11}H_{21}AlBr_2Si_2$: C 33.34 H 5.34; Found: C 32.37 H 5.21.

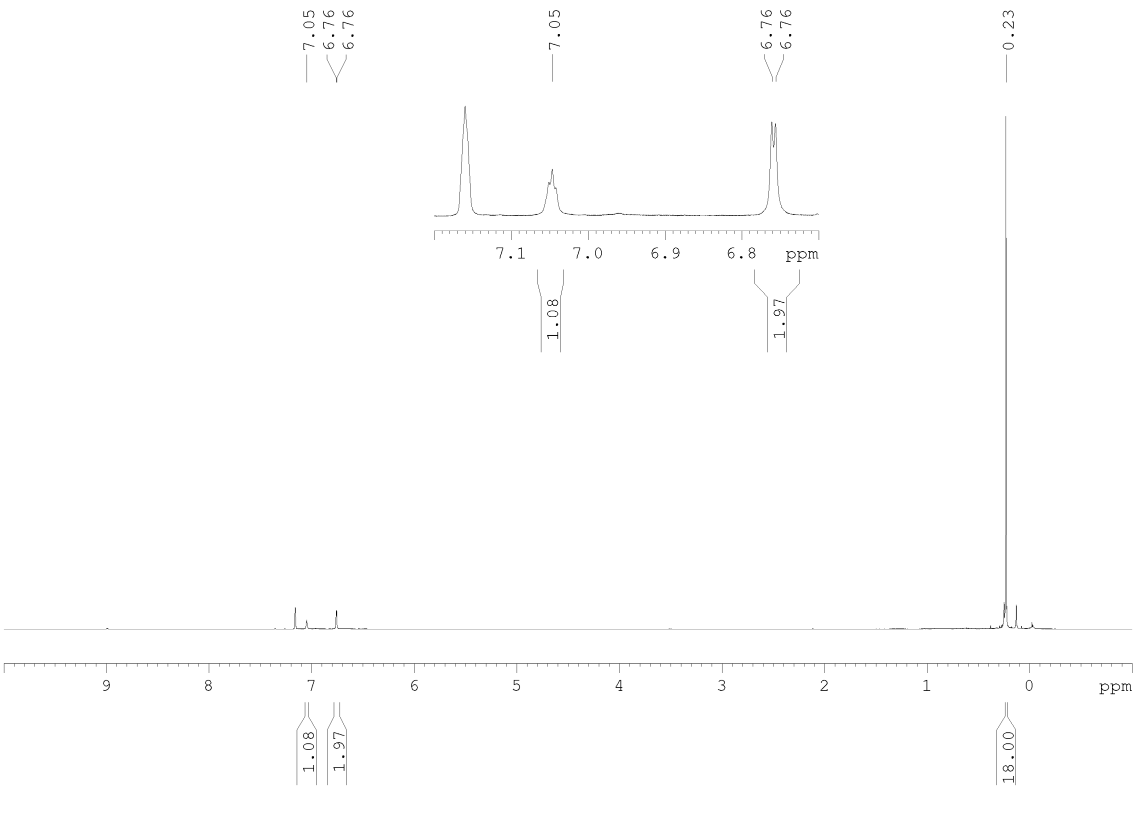

Fig. S1 | ^{1}H NMR spectrum of 2a in C_6D_6.

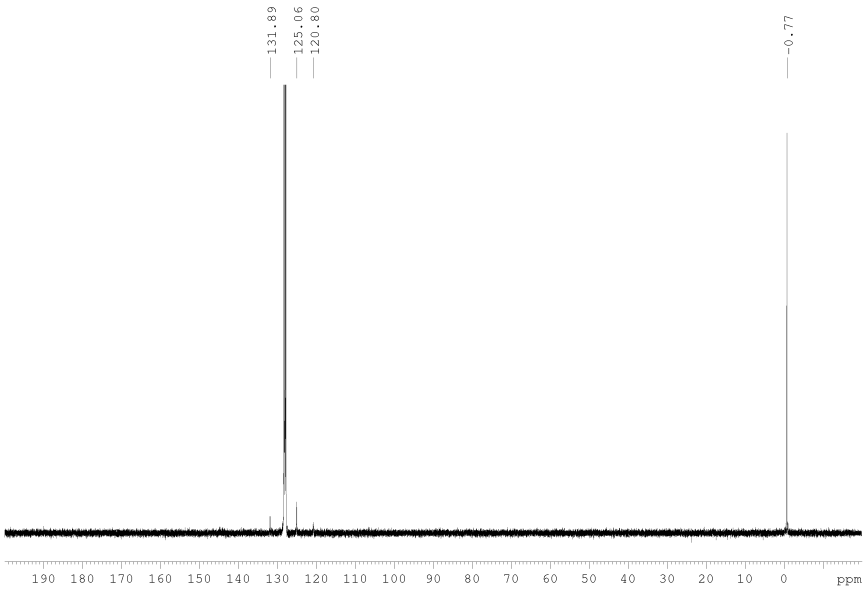

Fig. S2 | ^{13}C NMR spectrum of 2a in C_6D_6.

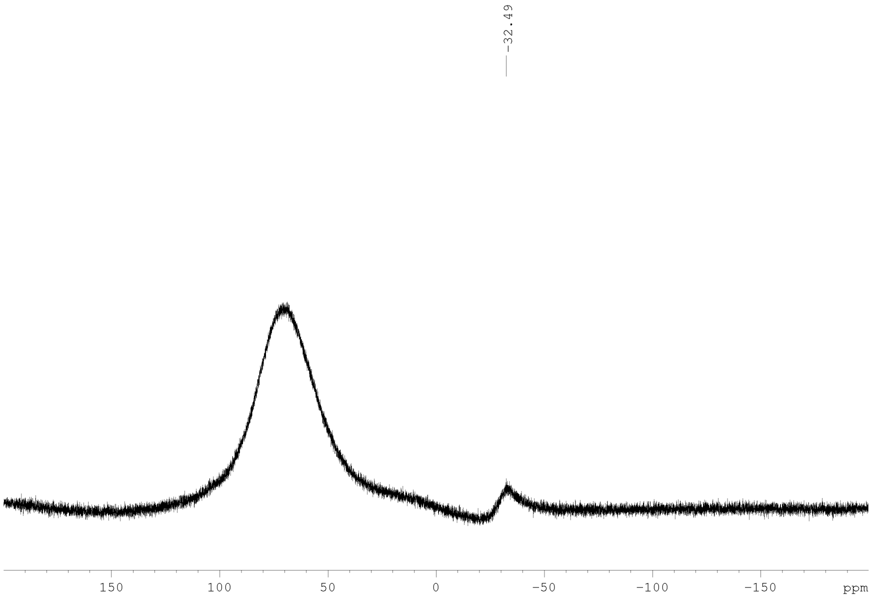

Fig. S3 | ^{27}Al NMR spectrum of 2a in C_6D_6.

Synthesis and characterization of $Cp^{3Si}AlBr_2$ 2b

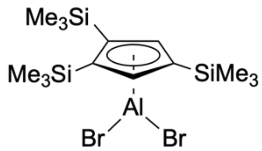

$(Cp^{3Si})_2Mg$ (250 mg, 425 μmol) was dissolved in benzene (70 mL), and $AlBr_3$ (226 mg, 850 μmol) was added. The solution turned yellow in colour, and a white precipitate formed immediately. After two hours at room temperature, the solution was filtered, the precipitate was washed twice with benzene (2 x 20 mL), and all volatiles were removed in vacuo. Extraction into pentane (50 mL), and crystallization at –30 °C afforded **2b** as a colourless, crystalline solid (312 mg, 704 μmol, 83%). Crystals suitable for X-Ray diffraction were obtained from saturated pentane solutions at –30 °C.

^{1}H NMR (400.1 MHz, C_6D_6): δ = 0.27 [s, 9H, $(Me_3Si)_3C_5H_2$], 0.38 [s, 18H, $(Me_3Si)_3C_5H_2$], 7.42 [s, 2H, $(Me_3Si)_3C_5H_2$].

^{13}C NMR (101 MHz, C_6D_6): δ = –0.70, 0.67 [s, $(Me_3Si)_3C_5H_2$], 121.27, 130.51, 137.95 [s, $(Me_3Si)_3C_5H_2$].

^{27}Al NMR (104 MHz, C_6D_6): δ = –39.

Elemental analysis calculated for $C_{14}H_{29}AlBr_2Si_3$: C 35.90 H 6.24; Found: C 35.78 H 6.33.

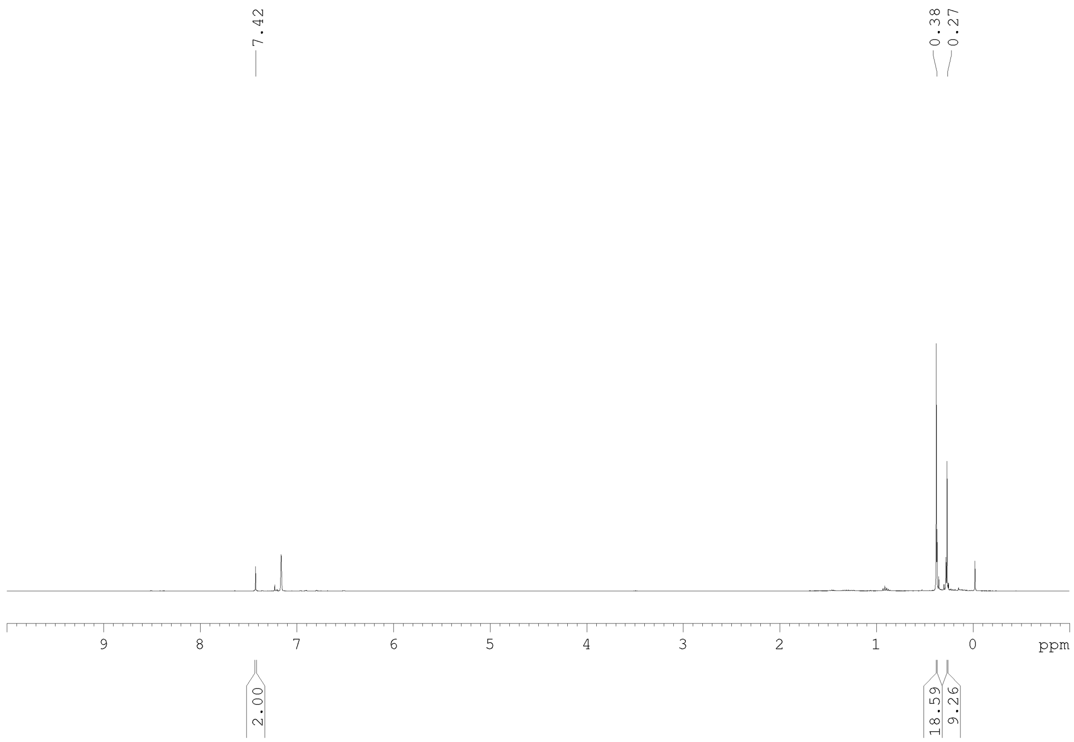

Fig. S4 | ^{1}H NMR spectrum of 2b in C_6D_6.

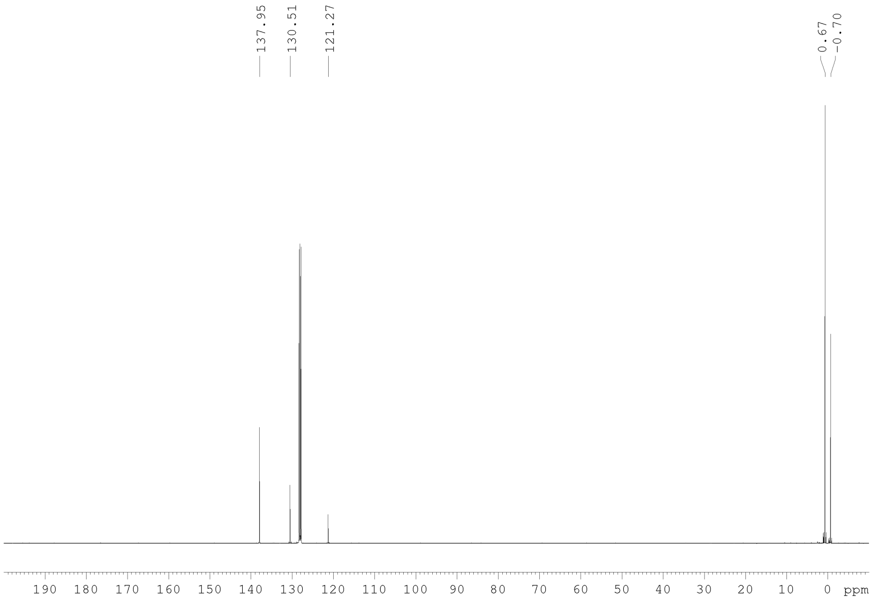

Fig. S5 | ^{13}C NMR spectrum of 2b in C_6D_6.

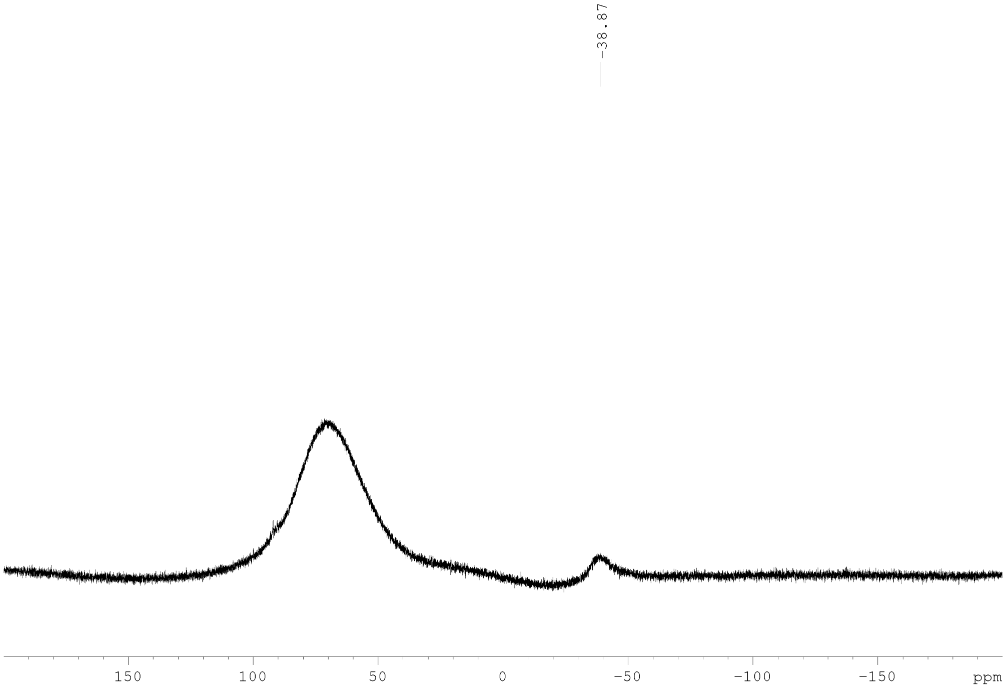

Fig. S6 | ^{27}Al NMR spectrum of 2b in C_6D_6.

Synthesis and characterization of 3a

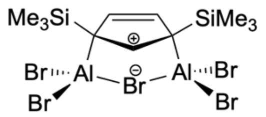

Method A (preferred): A solution of **2a** (500 mg, 1.26 mmol) in benzene (50 mL) was treated with $AlBr_3$ (336 mg, 1.26 mmol) and stirred at rt for 12 hours, during which time the pale-yellow colour of the reaction mixture intensified. Insolubilities were removed by filtration, and washed twice with pentane (2 x 10 mL). Removal of all volatiles in vacuo afforded **3a** as a colourless solid (348 mg, 524 μmol, 42%). Crystals suitable for X-Ray diffraction were obtained from saturated benzene solutions. **Method B:** $(Cp^{2Si})_2Mg$ (500 mg, 1.13 mmol) was dissolved in benzene (100 mL), and $AlBr_3$ (1.20 g, 4.51 mmol) was added. The pale-yellow mixture darkened slightly upon stirring at rt for 12 hours. Insolubilities were removed by filtration, and washed twice with pentane (2 x 20 mL). Removal of all volatiles in vacuo afforded **3a** as a colourless solid (410 mg, 618 μmol, 27%).

^{1}H NMR (400.1 MHz, C_6D_6): δ = 0.14 [s, 18H, $(Me_3Si)_2C_5H_3$], 7.06 [d, 2H, $(Me_3Si)_2C_5H_3$, $^3J_{HH}$ = 1.8 Hz], 9.00 [t, 1H, $(Me_3Si)_2C_5H_3$, $^3J_{HH}$ = 1.8 Hz].

^{13}C NMR (101 MHz, C_6D_6): δ = 1.94 [s, $(Me_3Si)_2C_5H_3$], 106.68, 144.79, 192.87 [s, $(Me_3Si)_2C_5H_3$].

^{27}Al NMR (104 MHz, C_6D_6): δ = 102.

Elemental analysis calculated for $C_{11}H_{21}Al_2Br_5Si_2$: C 19.93 H 3.19; Found: C 20.53 H 3.36.

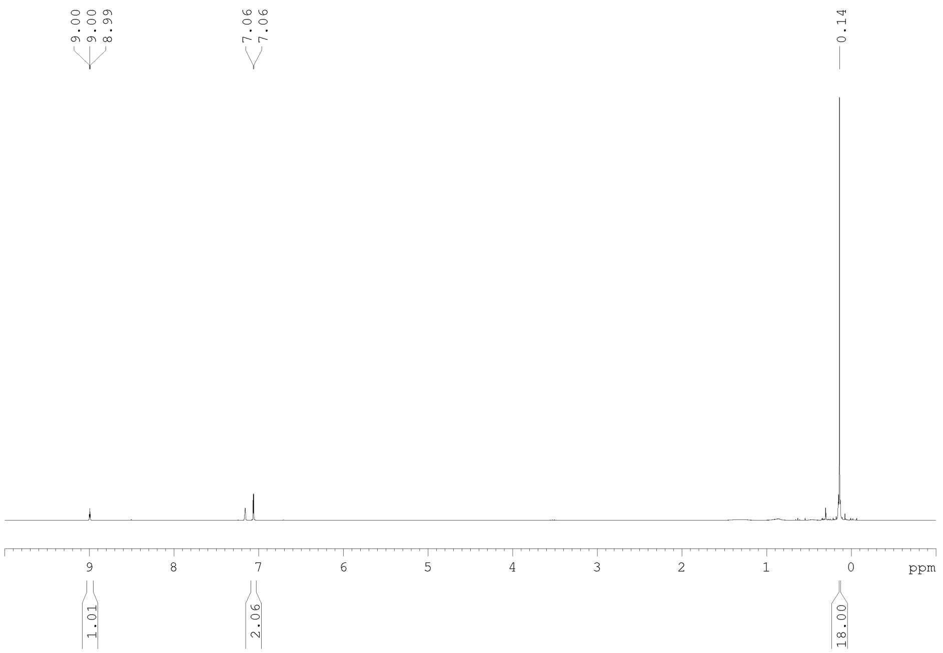

Fig. S7 | ^{1}H NMR spectrum of 3a in C_6D_6.

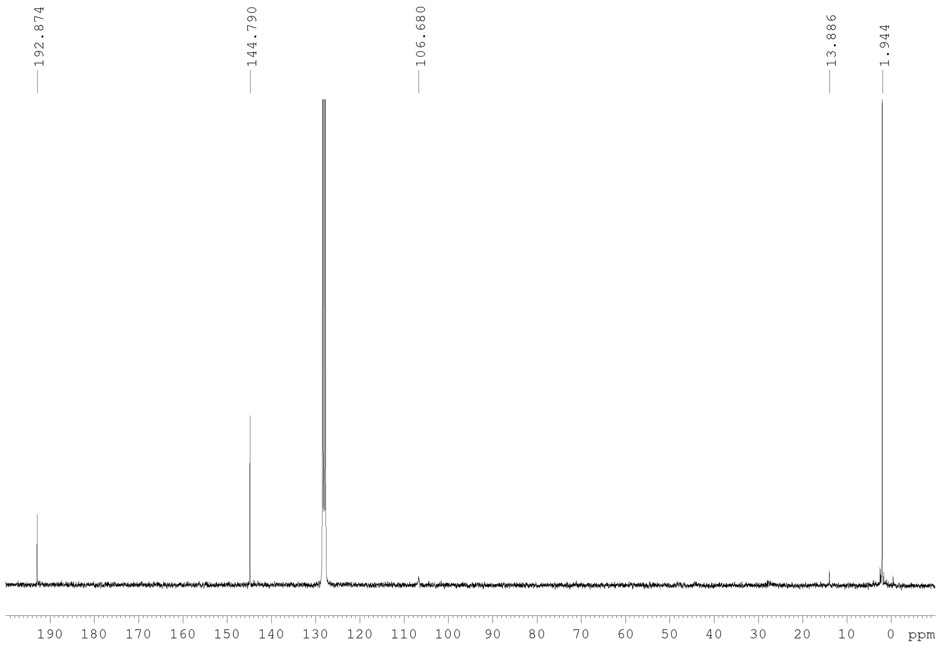

Fig. S8 | ^{13}C NMR spectrum of 3a in C_6D_6.

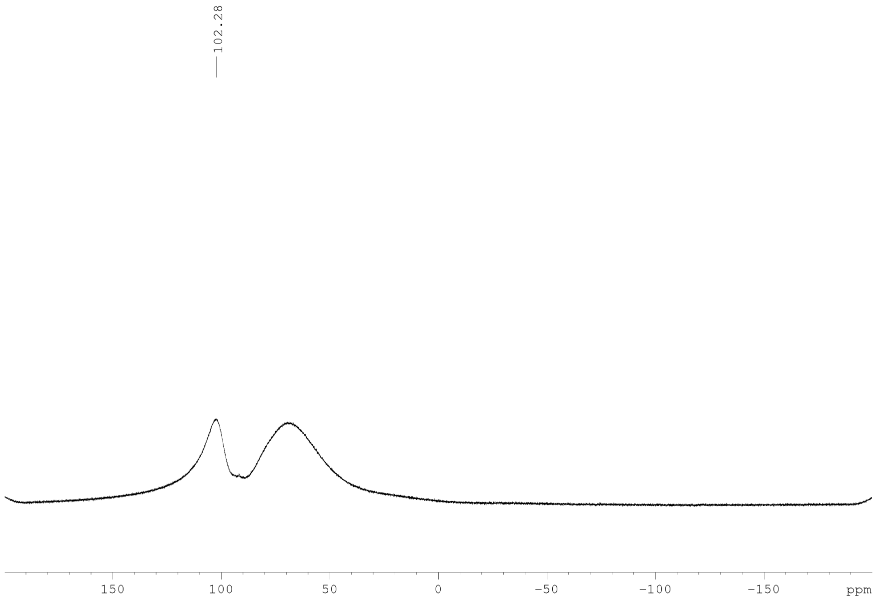

Fig. S9 | ^{27}Al NMR spectrum of 3a in C_6D_6.

Synthesis and characterization of 3b

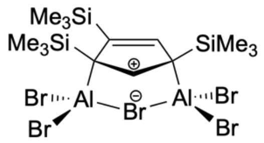

Method A (preferred): A solution of **2b** (500 mg, 1.07 mmol) in benzene (100 mL) was treated with $AlBr_3$ (285 mg, 1.07 mmol), and stirred at room temperature for two hours, during which time the pale-yellow colour of the reaction mixture intensified. Insolubilities were removed by filtration, and washed twice with pentane (2 x 20 mL). Removal of all volatiles in vacuo afforded **3b** as a colourless solid (421 mg, 559 μmol, 52%). Crystals suitable for X-Ray diffraction were obtained from saturated benzene solutions. **Method B:** $(Cp^{3Si})_2Mg$ (500 mg, 851 μmol) was dissolved in benzene (100 mL), and $AlBr_3$ (907 mg, 3.41 mmol) was added. The pale-yellow mixture darkened slightly upon stirring at room temperature for two hours. Insolubilities were removed by filtration, and washed twice with pentane (2 x 20 mL). Removal of all volatiles in vacuo afforded **3b** as a colourless solid (370 mg, 503 μmol, 30%).

^{1}H NMR (400.1 MHz, C_6D_6): δ = 0.15 [s, 9H, $(\mathit{Me}_3Si)_3C_5H_2$], 0.30 [s, 18H, $(\mathit{Me}_3Si)_3C_5H_2$], 8.50 [s, 2H, $(Me_3Si)_3C_5\mathit{H}_2$].

^{13}C NMR (101 MHz, C_6D_6): δ = 1.90, 2.44 [s, $(\mathit{Me}_3Si)_3C_5H_2$], 105.29, 137.77, 176.82 [s, $(Me_3Si)_3\mathit{C}_5H_2$].

^{27}Al NMR (104 MHz, C_6D_6): δ = 102.

^{1}H NMR (400.1 MHz, C_7D_8, 178 K): δ = 0.08 [s, 9H, $(\mathit{Me}_3Si)_3C_5H_2$], 0.19 [s, 9H, $(\mathit{Me}_3Si)_3C_5H_2$], 0.29 [s, 9H, $(\mathit{Me}_3Si)_3C_5H_2$], 7.60 [s, 1H, $(Me_3Si)_3C_5\mathit{H}_2$], 9.29 [s, 1H, $(Me_3Si)_3C_5\mathit{H}_2$].

^{13}C NMR (101 MHz, C_7D_8, 178 K): δ = 0.05, 0.35, 1.40 [s, $(\mathit{Me}_3Si)_3C_5H_2$], 103.55, 110.44, 154.86, 159.35, 196.39 [s, $(Me_3Si)_3\mathit{C}_5H_2$].

Elemental analysis calculated for $C_{14}H_{29}Al_2Br_5Si_2$: C 22.87 H 3.98; Found: C 22.61 H 4.12.

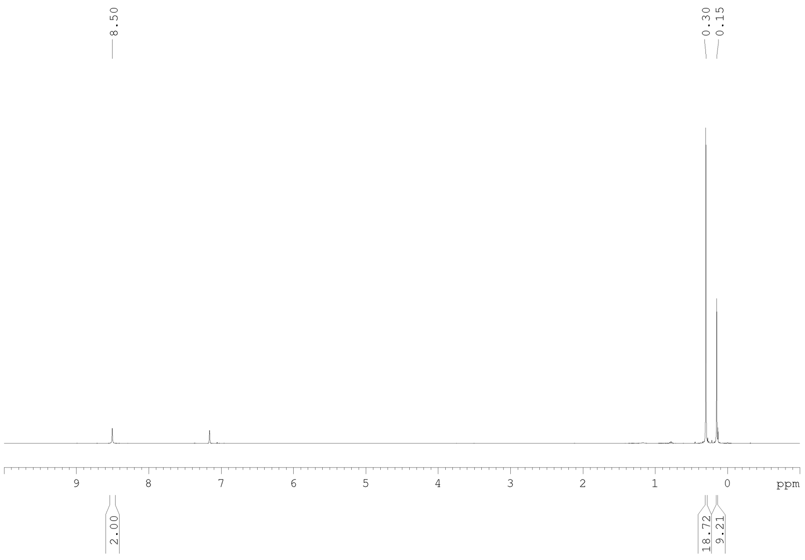

Fig. S10 | ^{1}H NMR spectrum of 3b in C_6D_6.

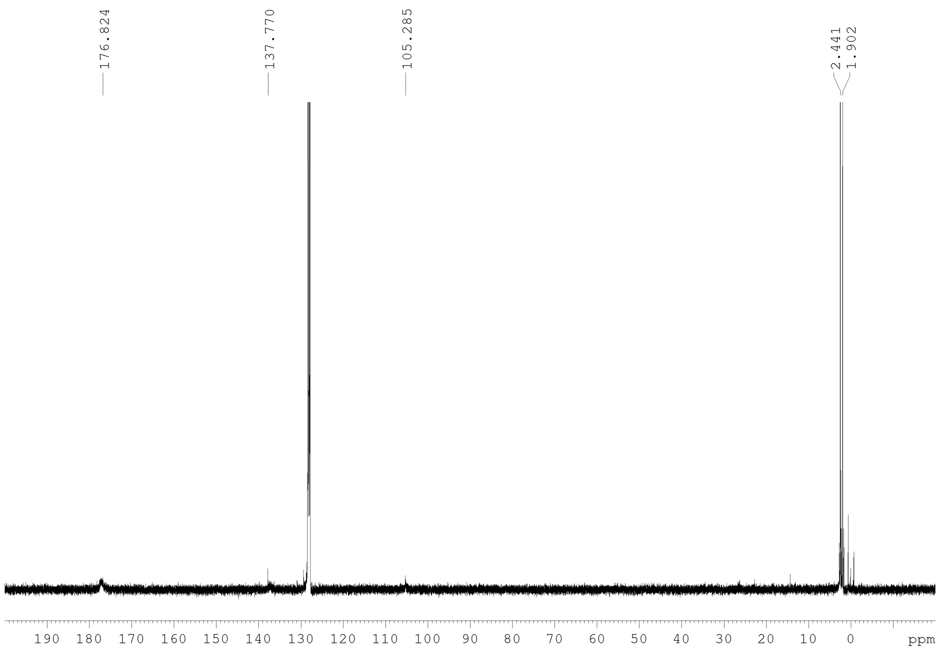

Fig. S11 | ^{13}C NMR spectrum of 3b in C_6D_6.

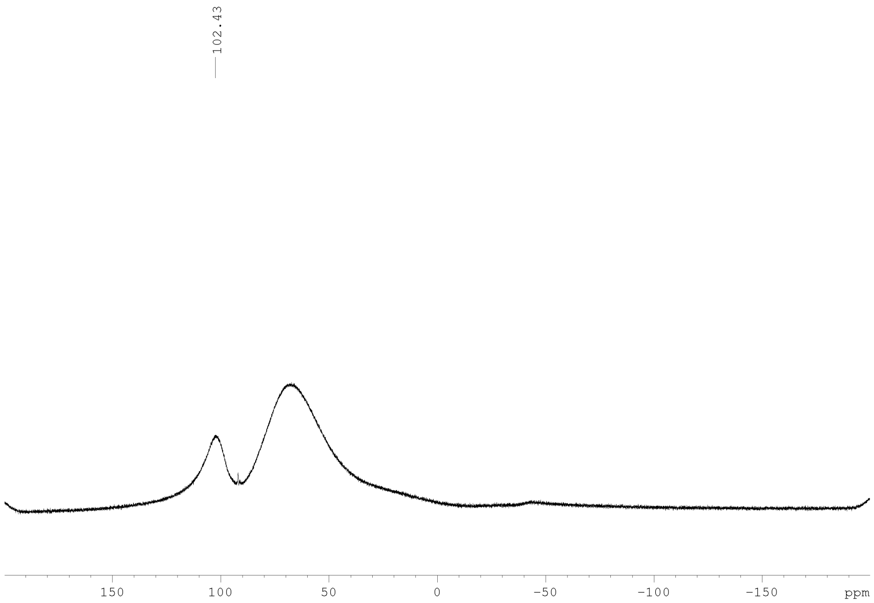

Fig. S12 | ^{27}Al NMR spectrum of 3b in C_6D_6.

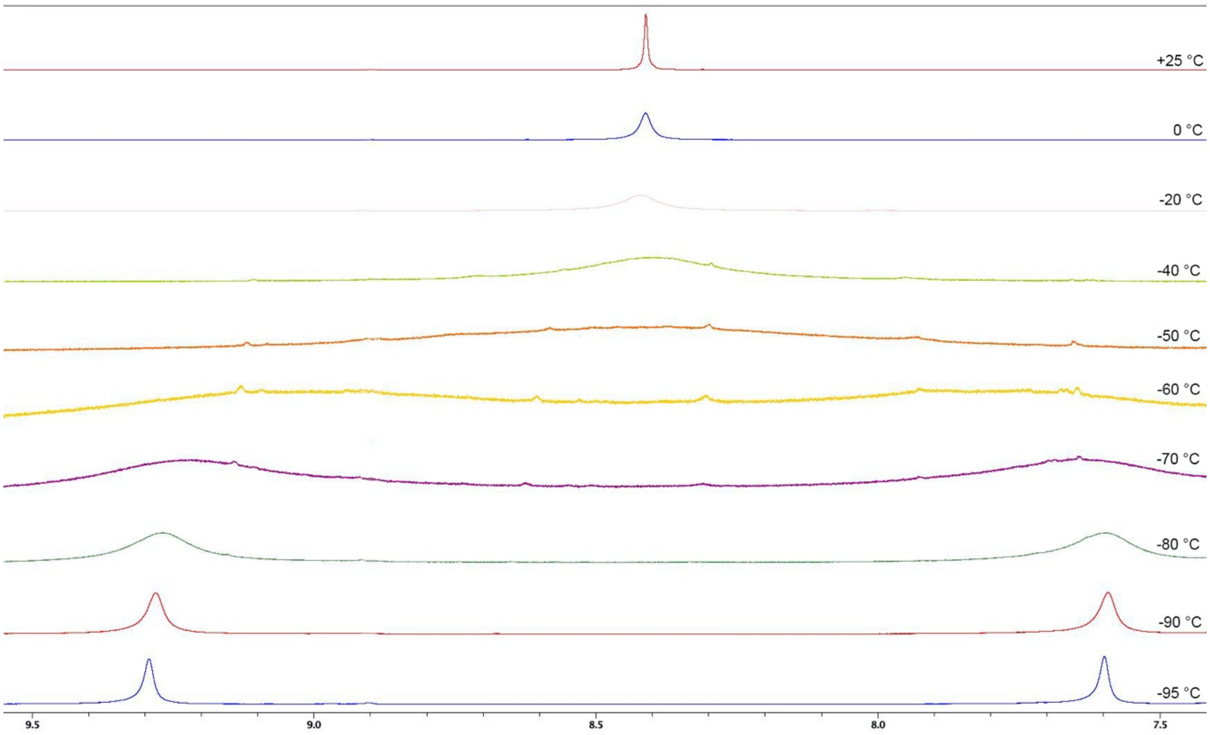

Fig. S13 | VT NMR spectroscopy on 3b in C_7D_8. T_C = 217 K, $\Delta\nu_0$ = 677.93 Hz, k_C = 1505 s^{-1}, $\Delta G^{\#}_C$ = 9.4±0.2 kcal/mol.

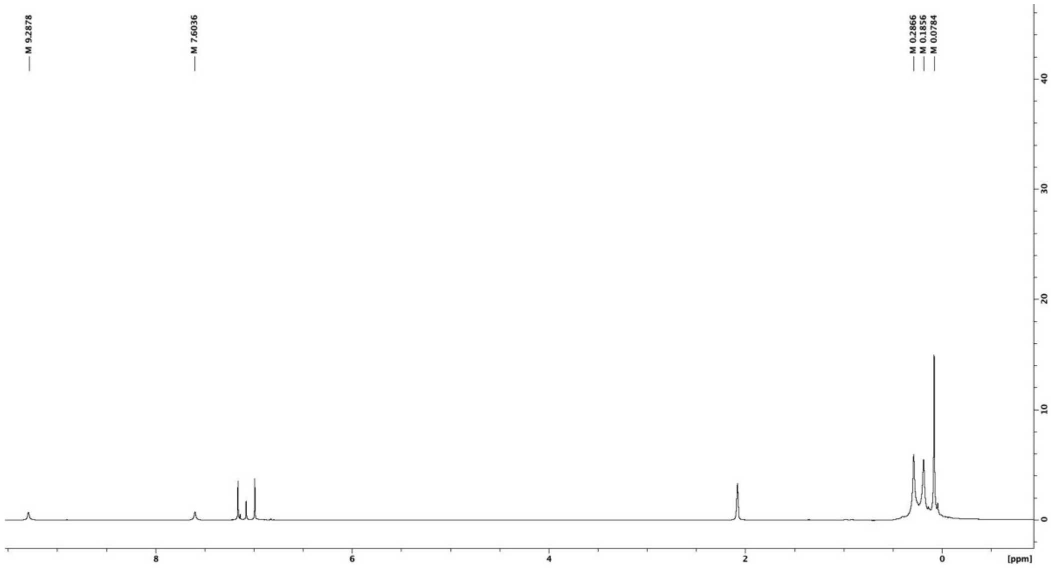

Fig. S14 | ^{1}H NMR spectrum of 3b in C_7D_8 at -95 °C.

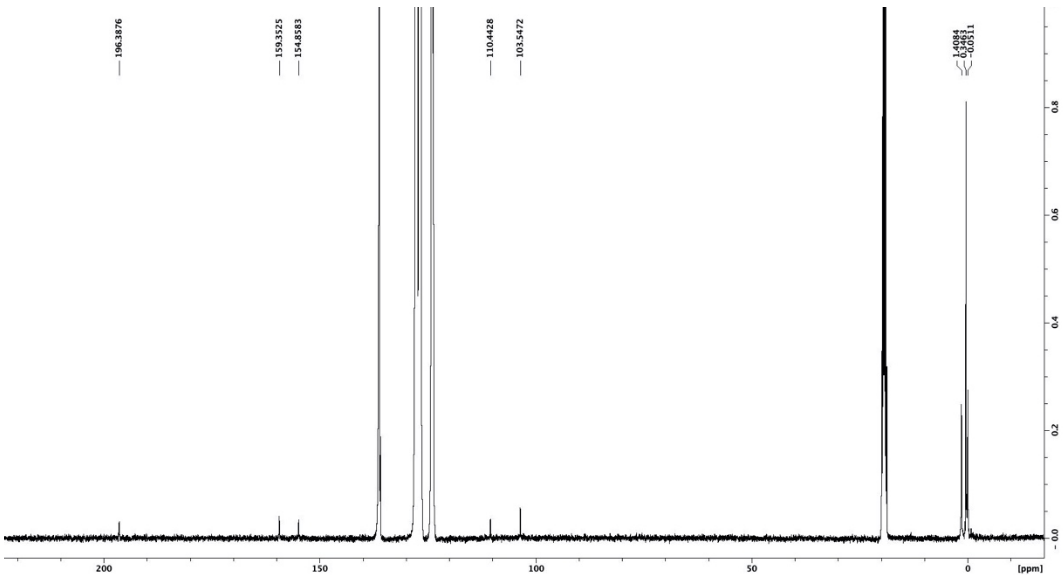

Fig. S15 |^{13}C NMR spectrum of 3b in C_7D_8 at -95 °C.

S2 X-ray diffraction data

General remarks: The crystal data of **2b**, **3a** and **2b** were collected on a BRUKER D8 QUEST diffractometer with a CMOS area detector and multi-layer mirror monochromated $Mo_{K\alpha}$ radiation. The structure was solved using intrinsic phasing method (SHELXT),[3] refined with the SHELXL program,[4] and expanded using Fourier techniques. All non-hydrogen atoms were refined anisotropically. Hydrogen atoms were included in structure factors calculations. All hydrogen atoms were assigned to idealized geometric positions.

Crystal data for **2b**: $C_{14}H_{29}AlBr_2Si_3$, M_r = 468.44, colorless block, 0.406×0.26×0.182 mm^3, Monoclinic space group *Cc*, *a* = 15.097(4) Å, *b* = 11.759(3) Å, *c* = 12.468(3) Å, β = 97.744(11)°, *V* = 2193.1(10) Å^3, *Z* = 4, ρ_{calcd} = 1.419 g·cm^{-3}, μ = 3.895 mm^{-1}, *F*(000) = 952, *T* = 100(2) K, R_1 = 0.0534, wR^2 = 0.1366, 4130 independent reflections [2θ≤52.044°] and 191 parameters.

Crystal data for **3a**: $C_{11}H_{21}Al_2Br_5Si_2$, M_r = 662.97, colorless block, 0.515×0.388×0.263 mm^3, Monoclinic space group *P*2$_1$/*c*, *a* = 21.6788(9) Å, *b* = 15.5211(6) Å, *c* = 14.0751(5) Å, β = 105.7320(10)°, *V* = 4558.6(3) Å^3, *Z* = 8, ρ_{calcd} = 1.932 g·cm^{-3}, μ = 8.988 mm^{-1}, *F*(000) = 2528, *T* = 100(2) K, R_1 = 0.0320, wR^2 = 0.0609, 8984 independent reflections [2θ≤52.044°] and 373 parameters.

Crystal data for **3b**: $C_{14}H_{29}Al_2Br_5Si_3$, M_r = 735.15, colorless block, 0.503×0.379×0.314 mm^3, Monoclinic space group *P*2$_1$/*n*, *a* = 9.067(2) Å, *b* = 17.002(6) Å, *c* = 17.752(6) Å, β = 91.435(18)°, *V* = 2735.8(14) Å^3, *Z* = 4, ρ_{calcd} = 1.785 g·cm^{-3}, μ = 7.550 mm^{-1}, *F*(000) = 1424, *T* = 100(2) K, R_1 = 0.0367, wR^2 = 0.0697, 5386 independent reflections [2θ≤52.042°] and 226 parameters.

Crystallographic data have been deposited with the Cambridge Crystallographic Data Center as supplementary publication no. CCDC1999909-1999911. These data can be obtained free of charge from The Cambridge Crystallographic Data Centre *via* www.ccdc.cam.ac.uk/data_request/cif.

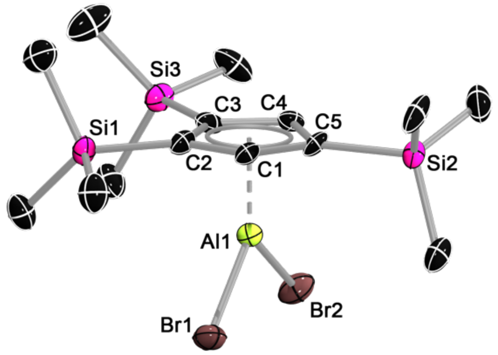

Fig. S16 | Molecular structure of 2b in the solid state. Thermal displacement parameters are displayed at the 50%-probability level. Hydrogen atoms are omitted for clarity. Selected bond lengths (Å): C1-C2 1.433(11), C1-C5 1.440(16), C2-C3 1.454(11), C3-C4 1.451(11), C4-C5 1.434(11), Al1-C1 2.201(11), Al1-C2 2.198(11), Al1-C3 2.183(12), Al1-C4 2.199(11), Al1-C5 2.193(12), Si1-C2 1.897(11), Si2-C5 1.870(11), Si3-C3 1.889(12).

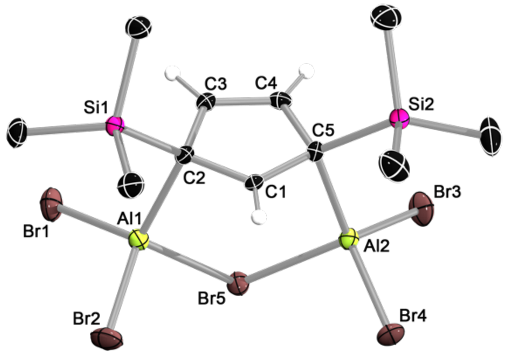

Fig. S17 | Molecular structure of 3a in the solid state. Thermal displacement parameters are displayed at the 50%-probability level. Most hydrogen atoms are omitted for clarity. The asymmetric unit contains two independent molecules; only one molecule is shown. Selected bond lengths C1-C2 1.435(4), C1-C5 1.424(4), C2-C3 1.477(4), C3-C4 1.347(4), C4-C5 1.479(4), Al1-C2 2.031(3), Al2-C5 2.038(3), Si1-C2 1.937(3), Si2-C5 1.943(3).

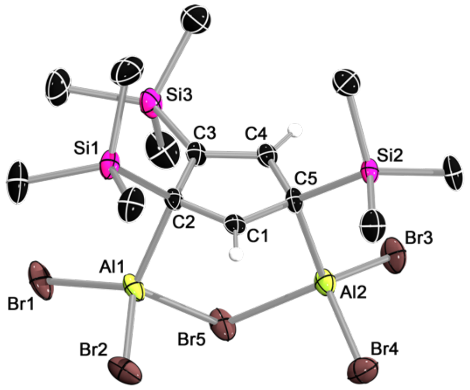

Fig. S18 | Molecular structure of 3b in the solid state. Thermal displacement parameters are displayed at the 50%-probability level. Most hydrogen atoms are omitted for clarity. Selected bond lengths (Å): C1-C2 1.427(4), C1-C5 1.426(4), C2-C3 1.492(4), C3-C4 1.376(4), C4-C5 1.469(4), Al1-C2 2.033(3), Al2-C5 2.043(3), Si1-C2 1.955(3), Si2-C5 1.939(3), Si3-C3 1.894(3).

S3 Computational details

General remarks: All computations were performed using the *Gaussian16* (Revision B.01) package.[5] All structures were fully optimized without symmetry constraints at the M06L level of theory employing Def2-SVP basis sets for all atoms.[6] This density-functional theory (DFT) method was chosen for the following reasons: (*i*) M06L has shown good performance in main-group and organometallic chemistry for assessing thermochemistry and kinetics;[7] (*ii*) As a local functional, M06L has comparatively low computational costs for large systems such as **3a**/**3b**; thus it represents a good compromise of accurancy and computational cost;[7] (*iii*) M06L has frequently been used successfully in our group for accurate calculations of various systems and parameters.[8-10] For all other calculations (single point energies, frequencies, nmr, nbo, nics, acid, orbitals), Def2-TZVP basis sets were used, combined with the SMD solvation model (*scrf=smd*) for inclusion of benzene solvent effects.[11] Zero-point vibrational energies and thermal corrections were computed from frequency calculations with a standard state of 298 K and 1 atm; both thermal free energies (ΔE_{298}), and Gibbs free energies (ΔG) were obtained from these single-point frequency calculations. The presence of true energy minima on the potential energy surface was verified for all optimized species by the absence of imaginary frequencies. Transition state geometries were obtained using *opt=(ts, noeigen,calcfc)* algorithms.[12] All optimized transition state structures were confirmed as maxima with only one imaginary frequency. Additionally, each transition state established was ensured to be on the preferred reaction path by performing "plus-and-minus-displacement" minimization calculations, which involved the displacement of transition state structures by ca. 0.05 Å along the imaginary frequency normal mode in both directions.[13,14] The displaced geometries were subsequently optimized to the nearest minimum.

Natural bond order (NBO) analysis and natural resonance theory (NRT) studies were carried out using the *NBO 7.0.2* program.[15] Intrinsic bonding orbitals (IBOs) were studied with the *IboView* program (v20150427).[16] Illustrations of optimized structures, and MOs were prepared with *GaussView6*.[17] The anisotropy of current (induced) density was studied employing the *AICD 3.0.2* software package.[18,19] Input files were generated with *Gaussian16* using the *nmr=csgt iop(10/93=1)* command. For separation of the ACID isosurfaces into π contributions, relevant molecular orbitals (MOs) were selected from suitable Gaussian output files, and included in ACID calculations *via* the *iop(10/93=2)* option of *Gaussian16.* Aromaticity descriptors were computed as follows: Multicenter delocalization indices (*mc*-DI)[20] and HOMA[21] (harmonic oscillator model of aromaticity) values were calculated using the *Multiwfn* program[22] employing *Gaussian16* formatted checkpoint files. Aromatic fluctuation indices (FLU),[23] I_{ring} values,[24] and multicenter indices (MCI)[25] were determined using the *ESI-3D* program.[26,27] Input files containing atomic overlap matrices were calculated with the *AIMAll 17.11.14* package employing Bader's QTAIM partitions.[28] All nucleus independent chemical shift calculations were performed according to the method developed by *Schleyer et al.* using the *Gaussian16* package.[29]

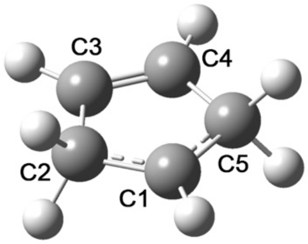

Fig. S19 | Optimized structure of 1^C. Selected bond lengths (Å): C1-C2 1.440, C1-C5 1.440, C2-C3 1.491, C3-C4 1.344, C4-C5 1.491.

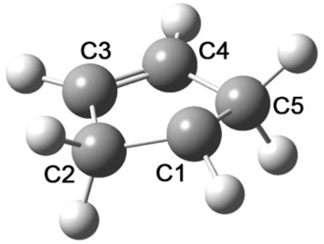

Fig. S20 | Optimized structure of $C_5H_7^-$. Selected bond lengths (Å): C1-C2 1.514, C1-C5 1.514, C2-C3 1.502, C3-C4 1.340, C4-C5 1.502.

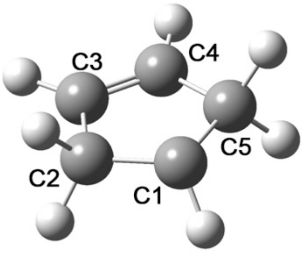

Fig. S21 | Optimized structure of $C_5H_7^-$, forced to planarity. Selected bond lengths (Å): C1-C2 1.486, C1-C5 1.506, C2-C3 1.534, C3-C4 1.344, C4-C5 1.504.

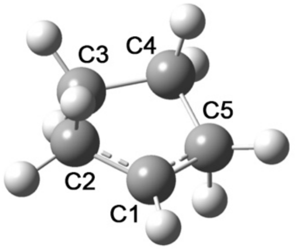

Fig. S22 | Optimized structure of $C_5H_9^+$. Selected bond lengths (Å): C1-C2 1.434, C1-C5 1.434, C2-C3 1.529, C3-C4 1.529, C4-C5 1.529.

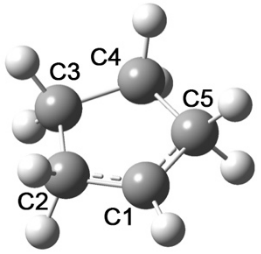

Fig. S23 | Optimized structure of $C_5H_9^+$, forced to planarity. Selected bond lengths (Å): C1-C2 1.426, C1-C5 1.447, C2-C3 1.524, C3-C4 1.545, C4-C5 1.523.

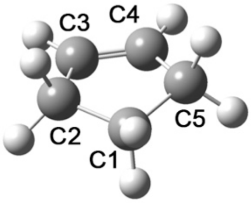

Fig. S24 | Optimized structure of C_5H_8. Selected bond lengths (Å): C1-C2 1.536, C1-C5 1.536, C2-C3 1.499, C3-C4 1.338, C4-C5 1.499.

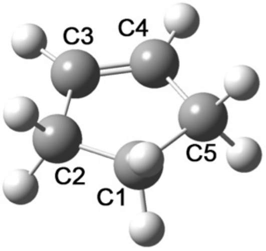

Fig. S25 | Optimized structure of C_5H_8, forced to planarity. Selected bond lengths (Å): C1-C2 1.491, C1-C5 1.544, C2-C3 1.549, C3-C4 1.342, C4-C5 1.497.

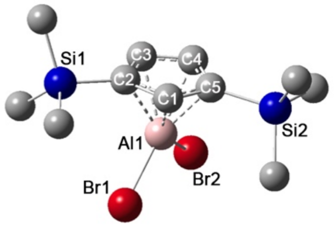

Fig. S26 | Optimized structure of 2a. Selected bond lengths (Å): av. C-C 1.430, av. Al-C 2.182.

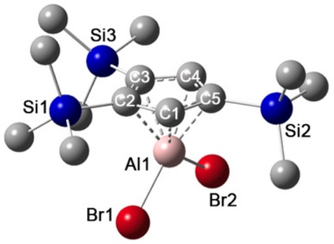

Fig. S27 | Optimized structure of 2b. Selected bond lengths (Å): av. C-C 1.435, av. Al-C 2.176.

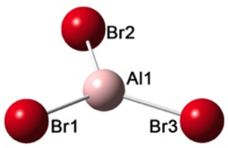

Fig. S28 | Optimized structure of $AlBr_3$. Selected bond lengths (Å): av. Al-Br 2.231.

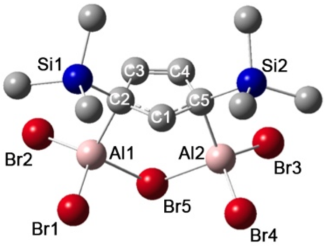

Fig. S29 | Optimized structure of 3a. Selected bond lengths (Å): C1-C2 1.427, C1-C5 1.427, C2-C3 1.469, C3-C4 1.366, C4-C5 1.468, Al1-C2 2.048, Al2-C5 2.053.

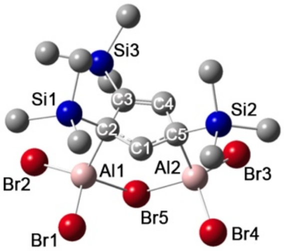

Fig. S30 | Optimized structure of 3b. Selected bond lengths (Å): C1-C2 1.432, C1-C5 1.423, C2-C3 1.484, C3-C4 1.377, C4-C5 1.464, Al1-C2 2.049, Al2-C5 2.050.

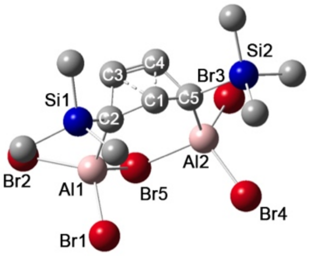

Fig. S31 | Optimized structure of $3a^B$. Selected bond lengths (Å): C1-C2 1.500, C1-C5 1.500, C2-C3 1.480, C3-C4 1.407, C4-C5 1.481, Al1-C2 2.000, Al2-C5 2.000, C1-C3 1.706, C1-C4 1.706.

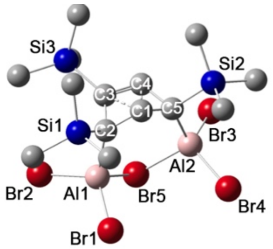

Fig. S32 | Optimized structure of $3b^B$. Selected bond lengths (Å): C1-C2 1.491, C1-C5 1.478, C2-C3 1.498, C3-C4 1.412, C4-C5 1.494, Al1-C2 2.000, Al2-C5 1.993, C1-C3 1.778, C1-C4 1.701.

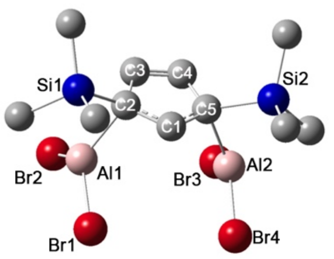

Fig. S33 | Optimized structure of 3a$^+$. Selected bond lengths (Å): C1-C2 1.426, C1-C5 1.426, C2-C3 1.466, C3-C4 1.362, C4-C5 1.466, Al1-C2 2.035, Al2-C5 2.035.

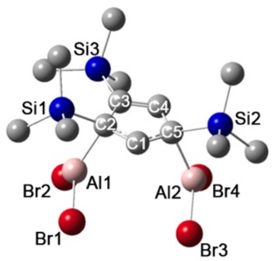

Fig. S34 | Optimized structure of 3b$^+$. Selected bond lengths (Å): C1-C2 1.429, C1-C5 1.424, C2-C3 1.484, C3-C4 1.375, C4-C5 1.464, Al1-C2 2.023, Al2-C5 2.027.

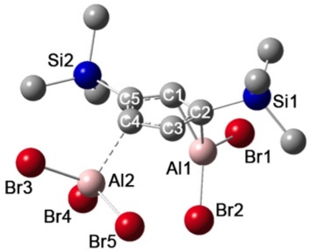

Fig. S35 | Optimized structure of TS1. Selected bond lengths (Å): C1-C2 1.490, C1-C5 1.408, C2-C3 1.416, C3-C4 1.416, C4-C5 1.432, Al1-C1 2.118, Al1-C2 2.046, Al2-C4 2.347.

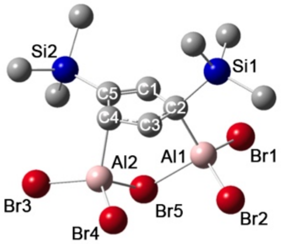

Fig. S36 | Optimized structure of INT. Selected bond lengths (Å): C1-C2 1.465, C1-C5 1.378, C2-C3 1.437, C3-C4 1.413, C4-C5 1.461, Al1-C2 2.028, Al2-C5 2.130.

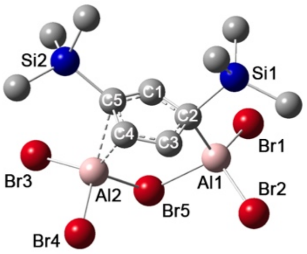

Fig. S37 | Optimized structure of TS2. Selected bond lengths (Å): C1-C2 1.452, C1-C5 1.398, C2-C3 1.447, C3-C4 1.391, C4-C5 1.469, Al1-C2 2.024, Al2-C4 2.227, Al2-C5 2.339.

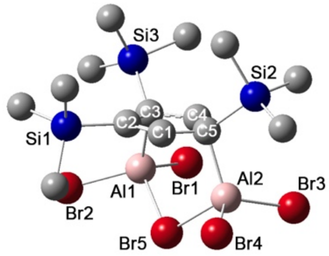

Fig. S38 | Optimized structure of 3b'. Selected bond lengths (Å): C1-C2 1.377, C1-C5 1.464, C2-C3 1.483, C3-C4 1.431, C4-C5 1.425, Al1-C3 2.051, Al2-C5 2.044.

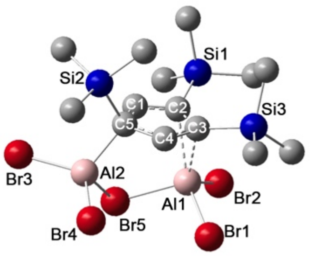

Fig. S39 | Optimized structure of TS$_{3b\text{-}3b'}$. Selected bond lengths (Å): C1-C2 1.396, C1-C5 1.449, C2-C3 1.487, C3-C4 1.404, C4-C5 1.449, Al1-C2 2.360, Al1-C3 2.241, Al2-C5 2.027.

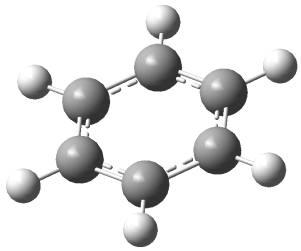

Fig. S40 | Optimized structure of C_6H_6. Selected bond lengths (Å): av. C-C 1.395.

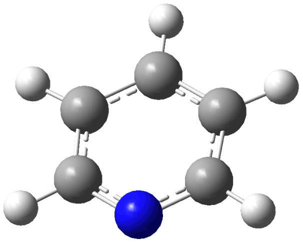

Fig. S41 | Optimized structure of NC_5H_5. Selected bond lengths (Å): av. C-C 1.394, av. C-N 1.333.

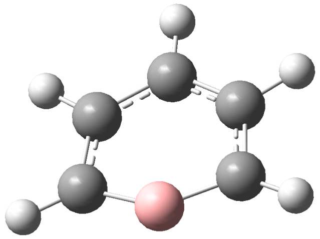

Fig. S42 | Optimized structure of BC_5H_5. Selected bond lengths (Å): av. C-C 1.405, B-C 1.436.

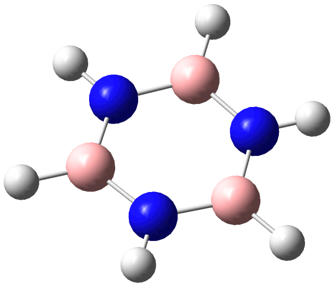

Fig. S43 | Optimized structure of $B_3N_3C_6$. Selected bond lengths (Å): av. B-N 1.427.

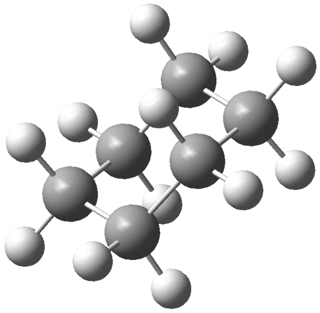

Fig. S44 | Optimized structure of C_6H_{12}. Selected bond lengths (Å): av. C-C 1.523.

Table S1 | Calculated parameters. Thermal free energies E_{298} (a.u.), and Gibbs free energies *G* (a.u.) of optimized structures. ACID critical isosurface values (CIVs, a.u.) for the central C_5 carbocyle.

	$\boldsymbol{E_{298}}$	***G***	**CIV**
$\mathbf{1^C}$	-194.397118	-194.429122	0.071
$C_5H_7^-$	-194.634833	-194.666158	0.039
$C_5H_7^-$ (fp)	-194.631685	-194.663979	-
$C_5H_9^+$	-195.606518	-195.638452	0.037
$C_5H_9^+$ (fp)	-195.601732	-195.635077	-
C_5H_8	-195.247137	-195.278145	0.036
C_5H_8 (fp)	-195.245021	-195.277635	-
**2a**	-6401.407515	-6401.482074	-
**2b**	-6810.012149	-6810.100182	-
$AlBr_3$	-7964.818136	-7964.856848	-
**3a**	-14366.259627	-14366.352758	0.060
**3b**	-14774.863048	-14774.969950	0.061
$\mathbf{3a^B}$	-14366.171256	-14366.264301	-
$\mathbf{3b^B}$	-14774.776568	-14774.884164	-
$\mathbf{3a^+}$	-11791.904980	-11791.993842	0.054
$\mathbf{3b^+}$	-12200.506552	-12200.611300	0.056
**TS1**	-14366.218751	-14366.311539	-
**INT**	-14366.254380	-14366.347568	-
**TS2**	-14366.245122	-14366.336481	-
**3b‘**	-14774.861904	-14774.968165	-
$\mathbf{TS_{3b \rightarrow 3b'}}$	-14.774.852544	-14.774.955712	-
$\mathbf{C_6H_6}$	-232.196835	-232.228554	-
$\mathbf{NC_5H_5}$	-248.249985	-248.281555	-
$\mathbf{BC_5H_5}$	-218.287076	-218.319127	-
$\mathbf{B_3N_3H_6}$	-242.622468	-242.655624	-
$\mathbf{C_6H_{12}}$	-235.754067	-235.787988	-

Table S2 | Aromaticity descriptors used to study electron delocalization in **1^C**, **3a**, **3b**, **3a⁺**, **3b⁺**, and selected reference molecules.

	NICS(1)$_{zz}$[a]	NICS(0)$_{\pi zz}$[a]	NICS(0)$_{\pi}$[a]	NICS(0)$_{iso}$[a]	HOMA	*mc*-DI[b]	FLU[b]	MCI[b]	I_{ring}[b]
**1^C**	-11.2	-20.9	-12.0	-2.3	-0.042	3.1	15.4	5.9	7.5
**3a**	-23.1	-25.0	-14.4	-5.7	0.48	5.6	14.4	33.2	24.9
**3b**	-22.7	-21.7	-11.0	-5.0	0.43	5.4	14.4	31.9	24.4
**3a⁺**	-20.4	-21.2	-10.4	-5.1	0.49	6.2	14.6	34.5	26.2
**3b⁺**	-19.7	-22.3	-10.9	-4.4	0.44	5.9	15.0	33.2	25.4
C_6H_6	-28.9	-36.7	-24.5	-8.3	1	20.5	0	72.1	48.1
NC_5H_6	-28.4	-36.6	-25.0	-7.1	0.97	19.8	4.1	66.0	44.7
BC_5H_6	-25.4	-33.0	-24.6	-11.9	0.99	17.4	-[c]	43.7	29.7
$B_3N_3H_6$	-6.3	-17.1	-14.3	-1.7	0.95	5.5	-[c]	2.0	1.6
C_6H_{12}	-	-	-	1.9	-2.47	0.1	91.8	0.3	0.3

[a] Given in ppm. [b] Values multiplied by 1000. With the exception of FLU, the degree of aromaticity rises with increasing indices. [c] No parameters implemented for B-C and B-N bonds.

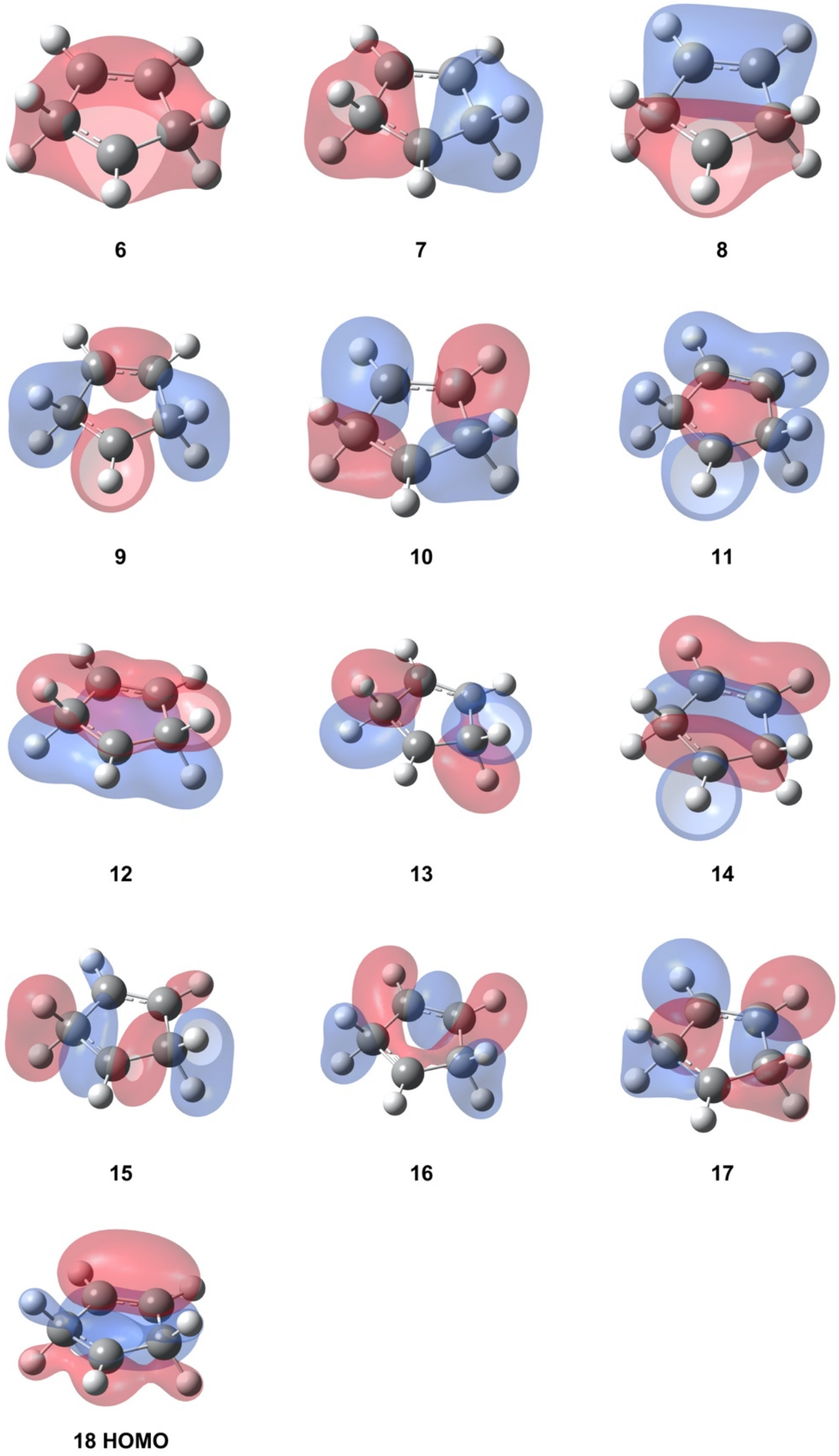

Fig. S45 | Selected Molecular orbitals of 1^C (Part 1). Isosurface plots at 0.04 a.u.

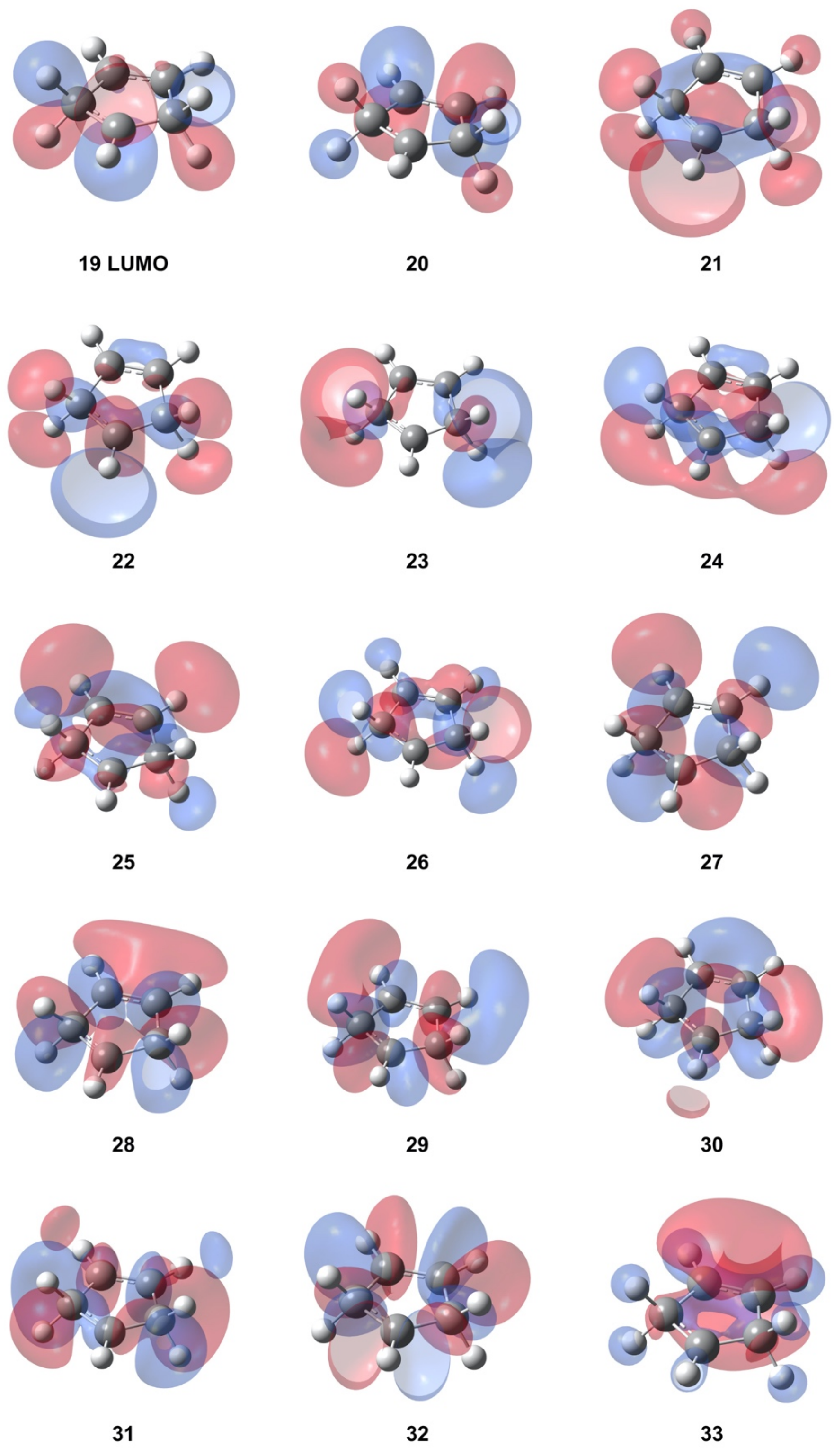

Fig. S46 | Selected Molecular orbitals of 1^{C} (Part 2). Isosurface plots at 0.04 a.u.

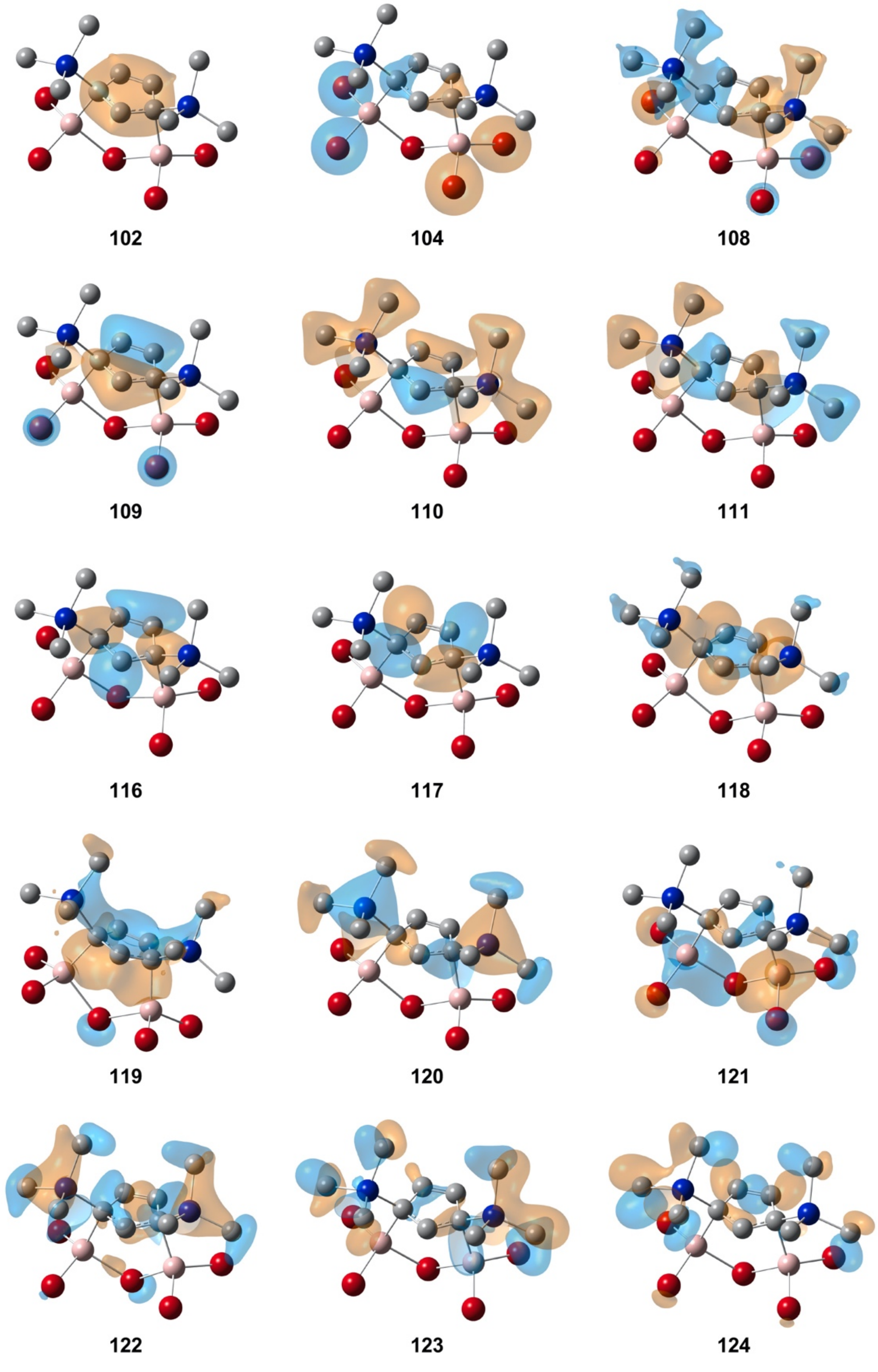

Fig. S47 | Selected Molecular orbitals of 3a (Part 1). Isosurface plots at 0.04 a.u.

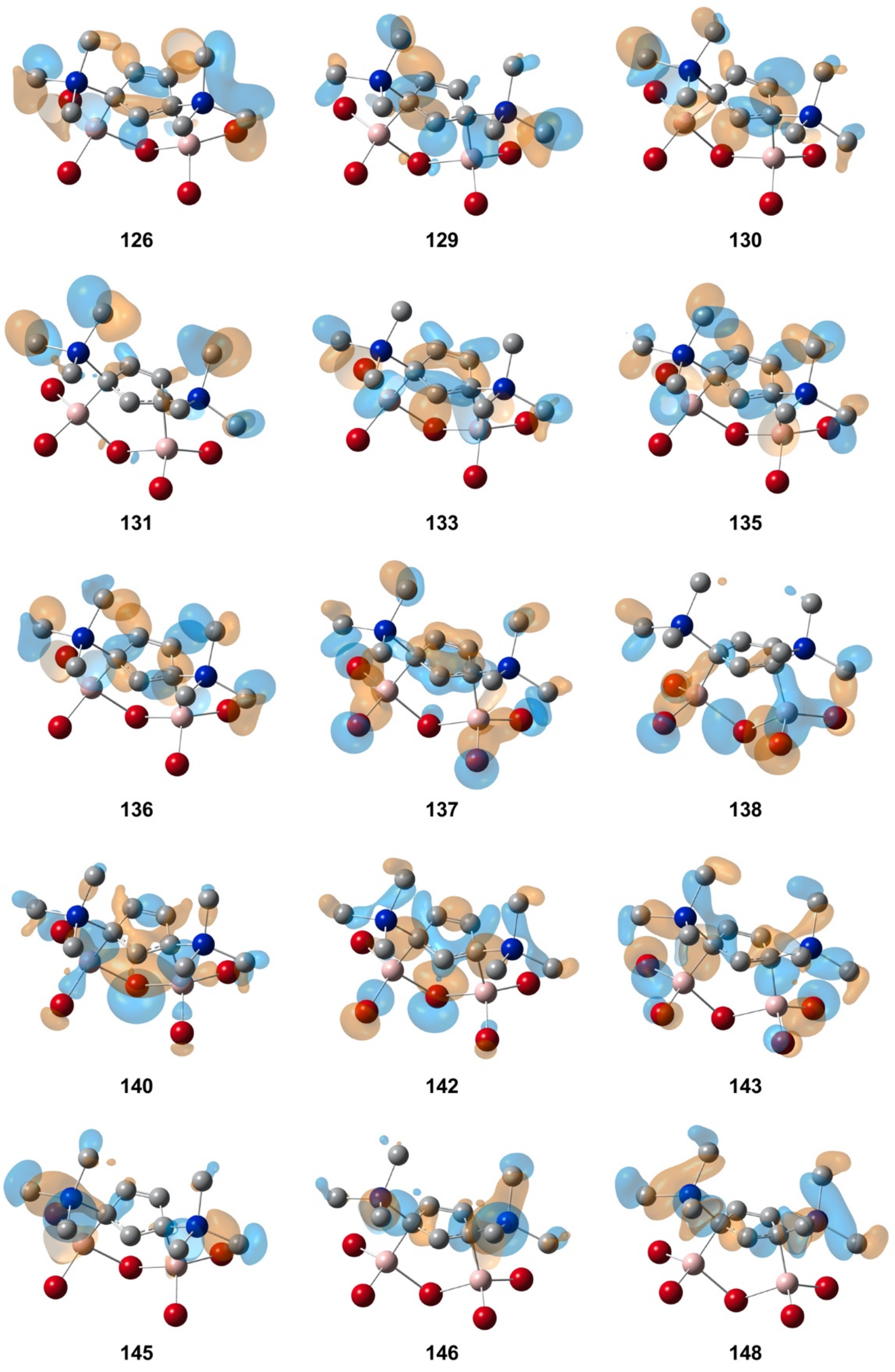

Fig. S48 | Selected Molecular orbitals of 3a (Part 2). Isosurface plots at 0.04 a.u.

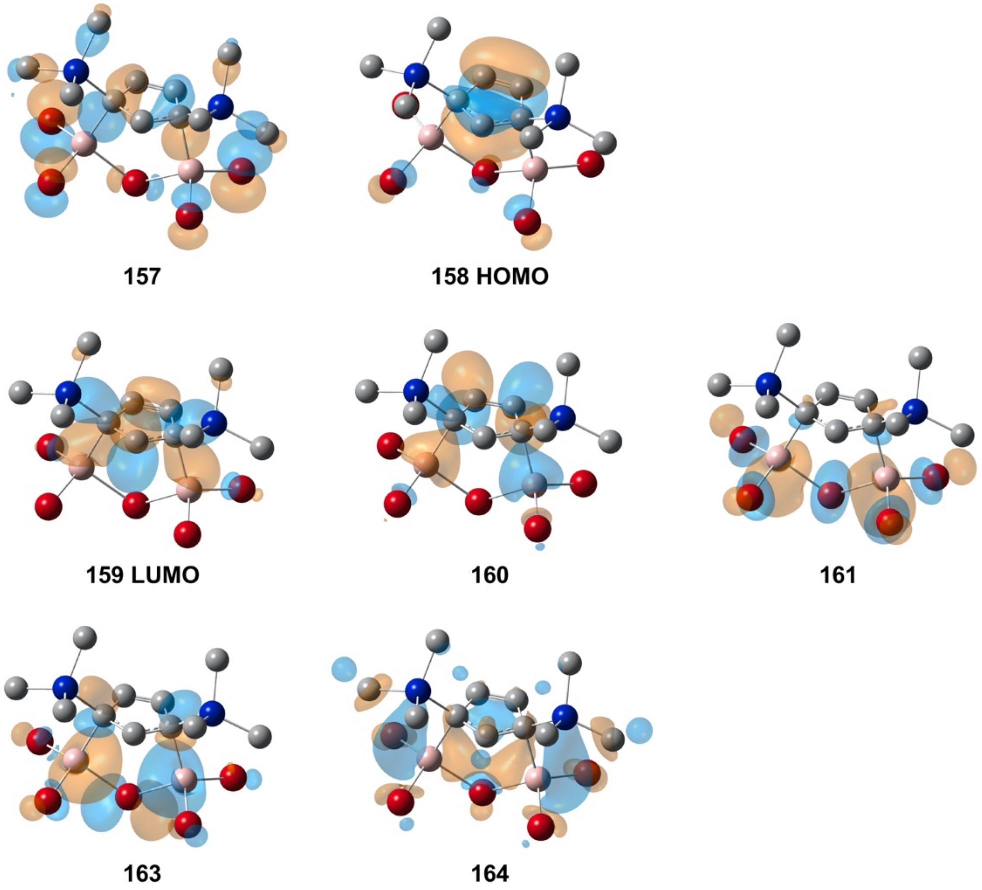

Fig. S49 | Selected Molecular orbitals of 3a (Part 3). Isosurface plots at 0.04 a.u.

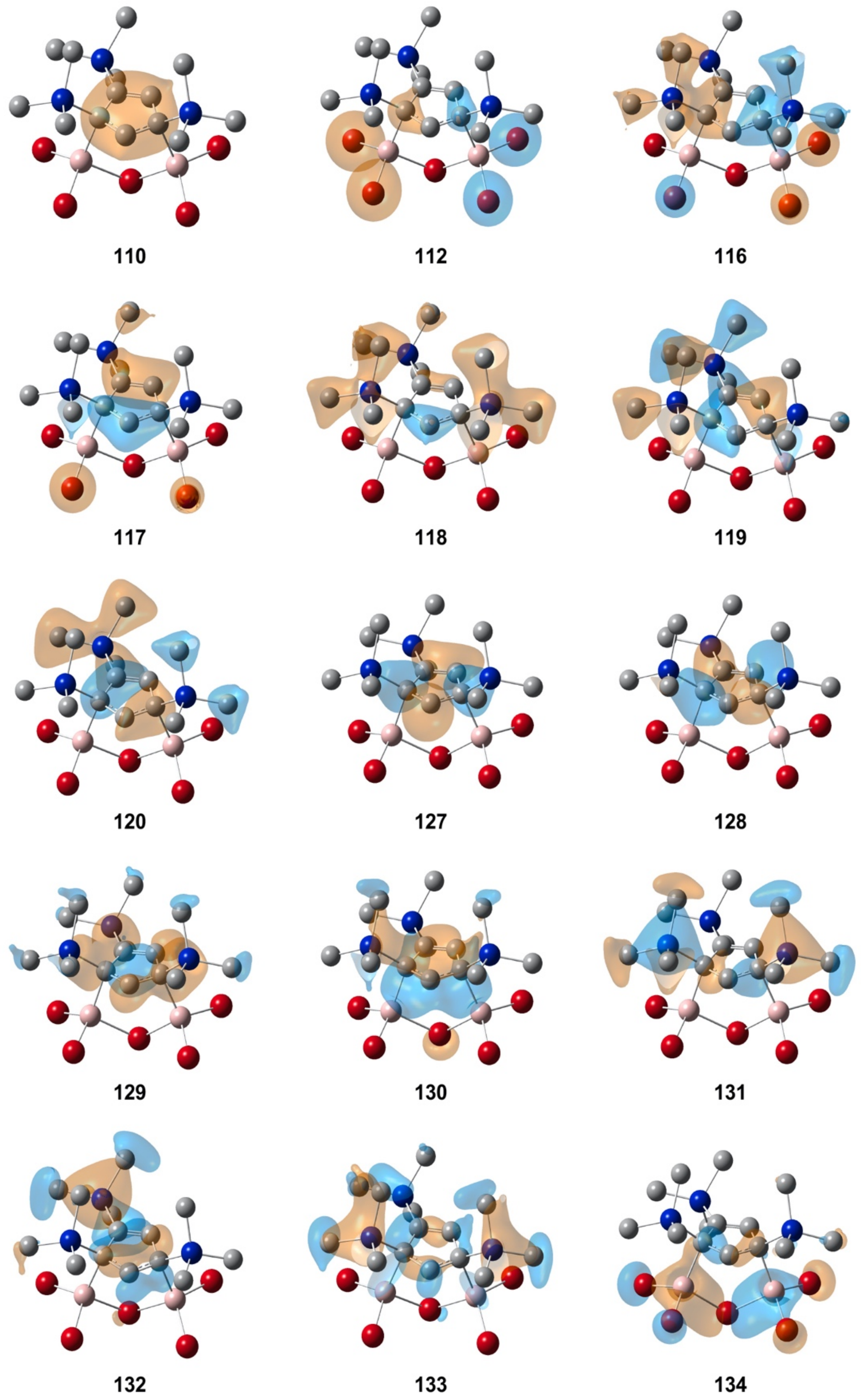

Fig. S50 | Selected Molecular orbitals of 3b (Part 1). Isosurface plots at 0.04 a.u.

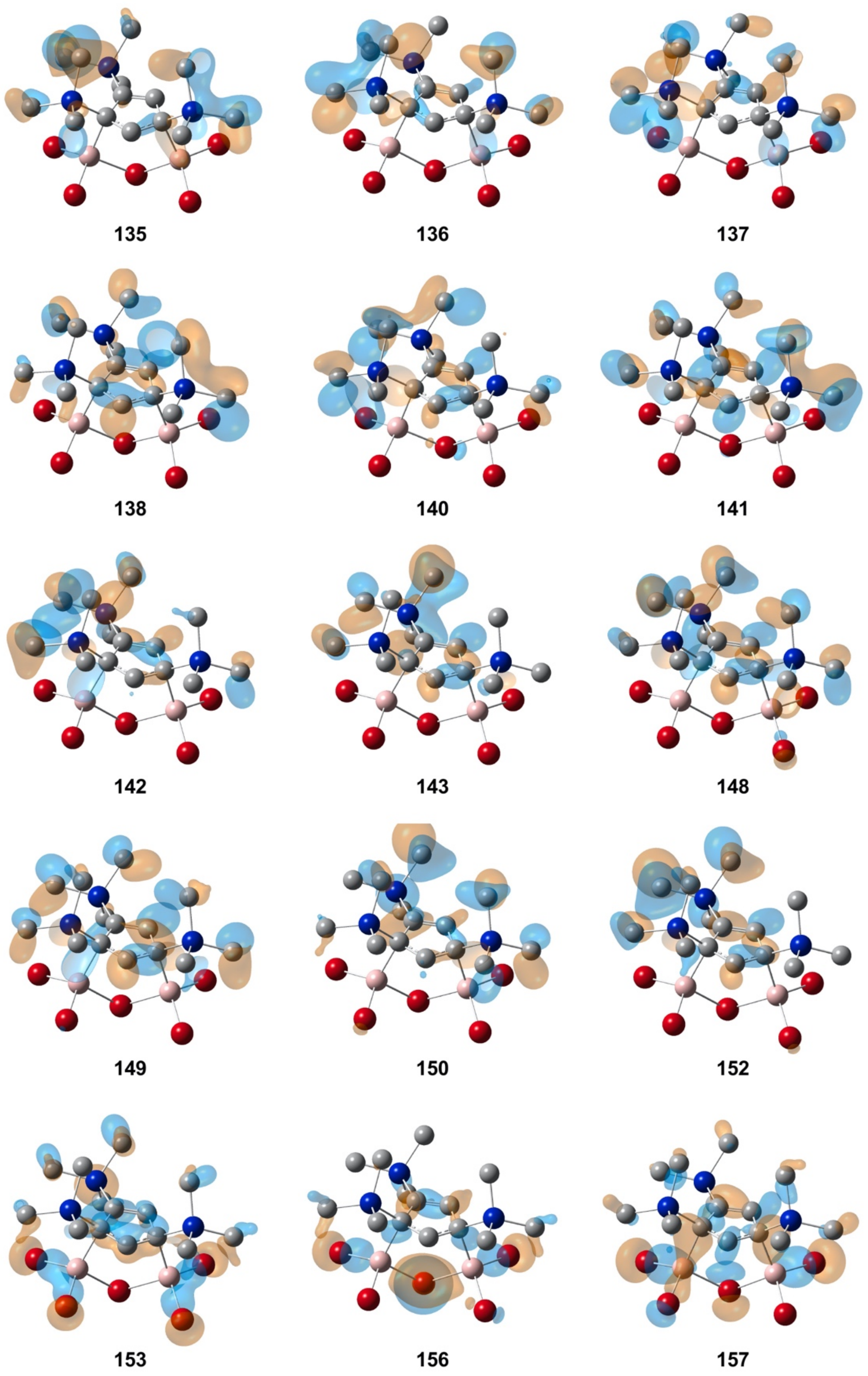

Fig. S51 | Selected Molecular orbitals of 3b (Part 2). Isosurface plots at 0.04 a.u.

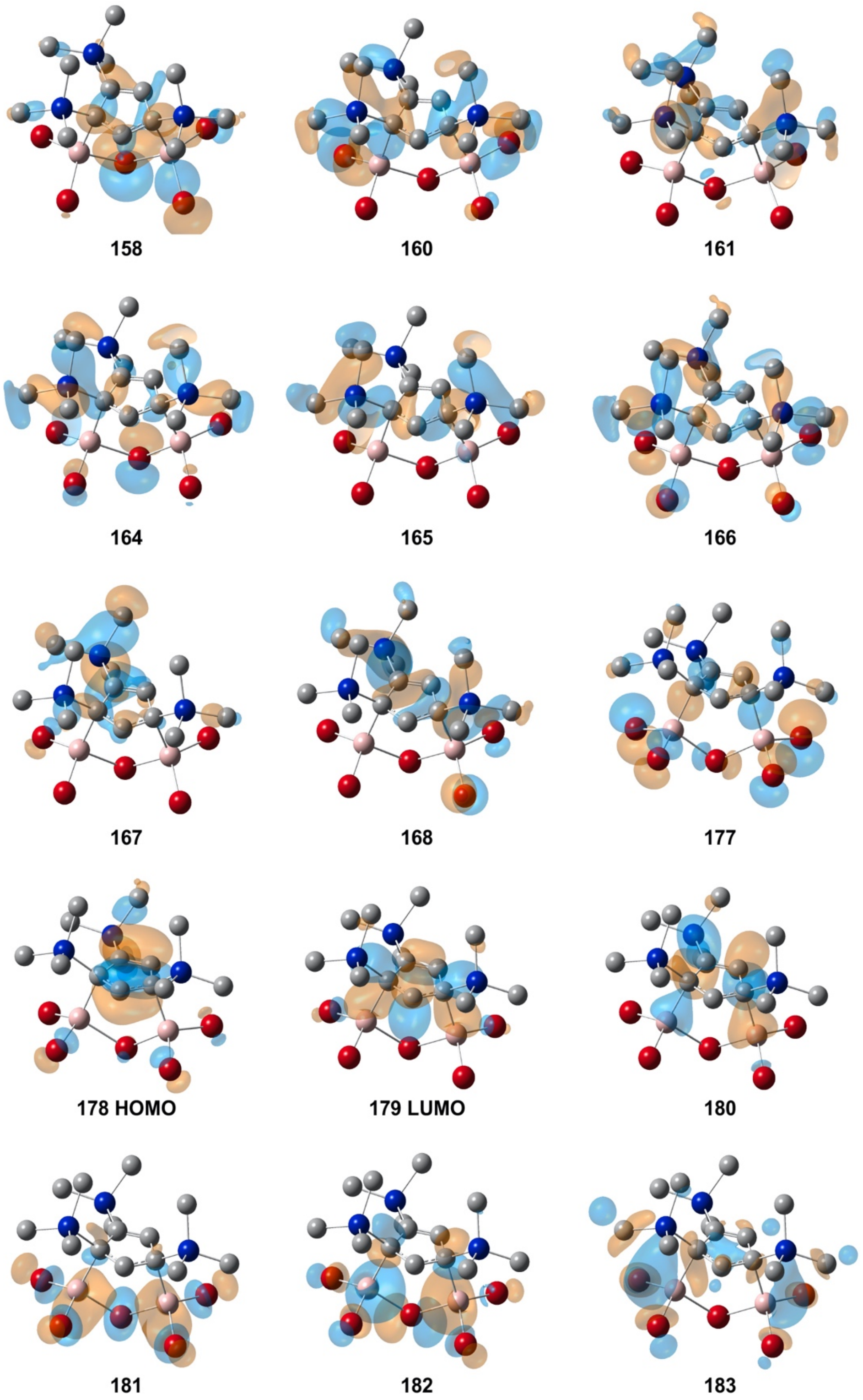

Fig. S52 | Selected Molecular orbitals of 3b (Part 3). Isosurface plots at 0.04 a.u.

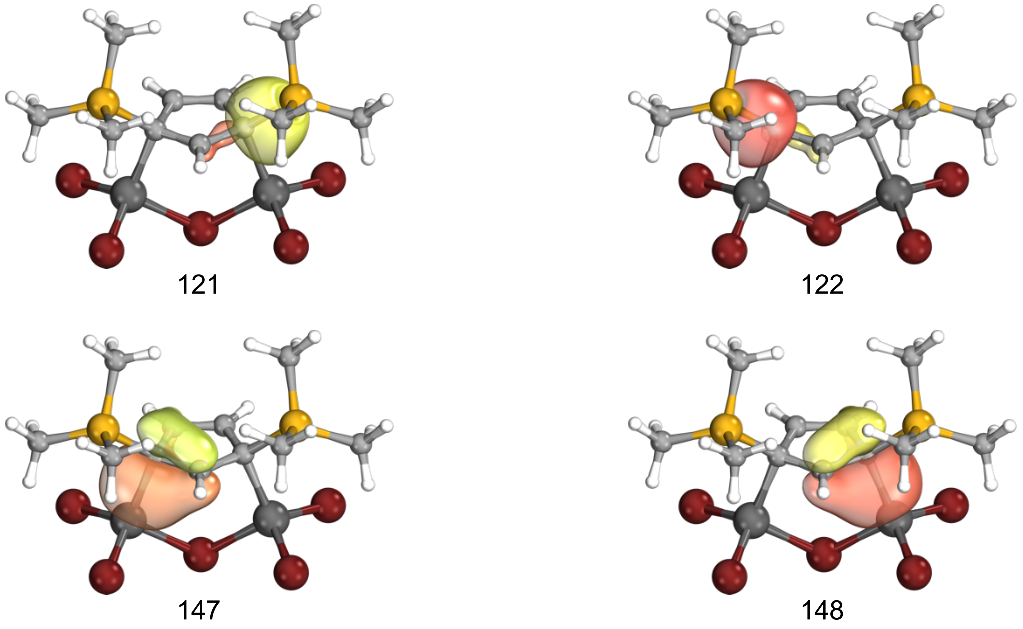

Fig. S53 | Selected intrinsic bonding orbitals of 3a relevant for hyperconjugation. The number of the respective IBO is given for each orbital.

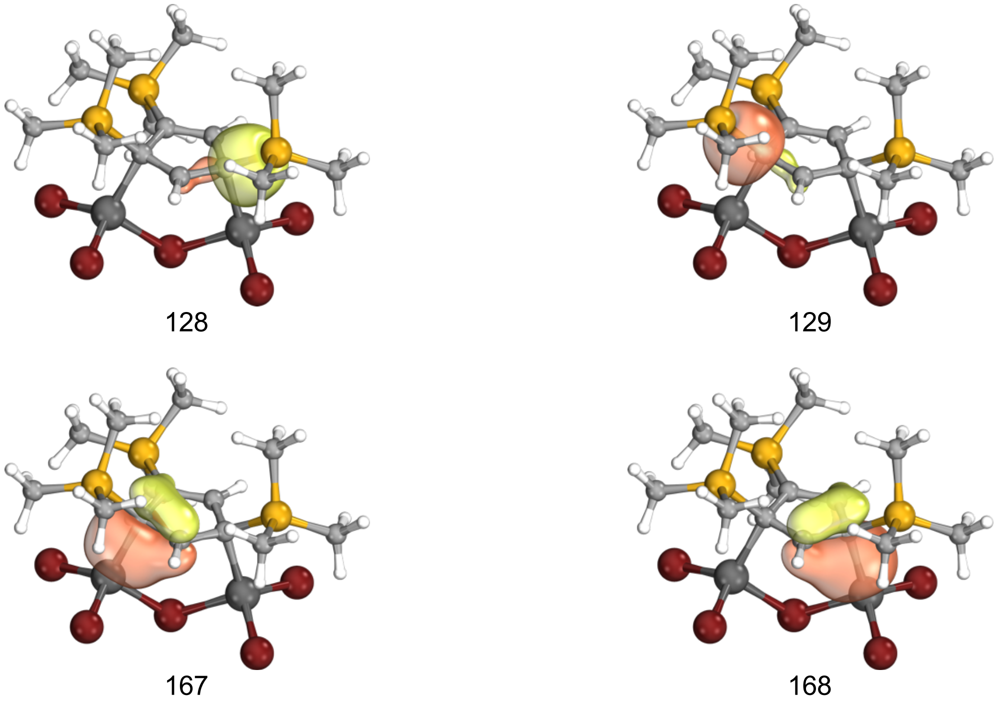

Fig. S54 | Selected intrinsic bonding orbitals of 3b relevant for hyperconjugation. The number of the respective IBO is given for each orbital.

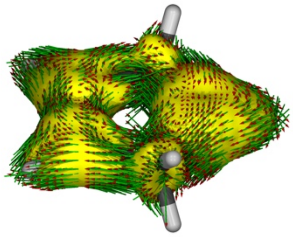

Fig. S55 | Total ACID isosurface of 1^C at 0.05 a.u. Current density vectors are plotted onto the ACID isosurface to indicate diatropic ring currents. Magnetic field is orthogonal with respect to the C_5 ring plane.

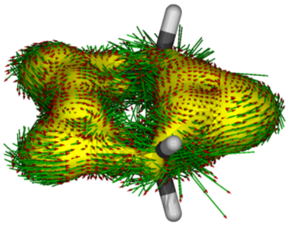

Fig. S56 | ACID isosurface of 1^C at 0.05 a.u. Involving MOs with mainly σ orbital contributions. Current density vectors are plotted onto the ACID isosurface to indicate paratropic ring currents. Magnetic field is orthogonal with respect to the C_5 ring plane.

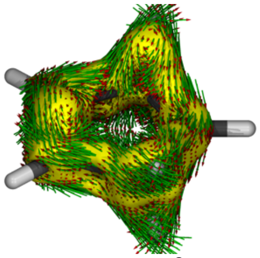

Fig. S57 | ACID isosurface of 1^C at 0.05 a.u. Involving MOs with mainly π orbital contributions. Current density vectors are plotted onto the ACID isosurface to indicate diatropic ring currents. Magnetic field is orthogonal with respect to the C_5 ring plane.

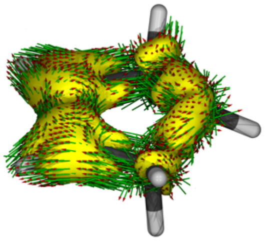

Fig. S58 | Total ACID isosurface of $C_5H_7^-$ at 0.055 a.u. Current density vectors are plotted onto the ACID isosurface to indicate diatropic ring currents. Magnetic field is orthogonal with respect to the C_5 ring plane.

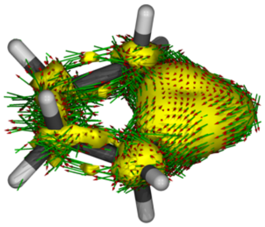

Fig. S59 | Total ACID isosurface of $C_5H_9^+$ at 0.055 a.u. Current density vectors are plotted onto the ACID isosurface to indicate diatropic ring currents. Magnetic field is orthogonal with respect to the C_5 ring plane.

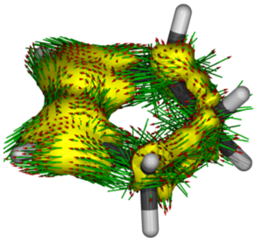

Fig. S60 | Total ACID isosurface of C_5H_8 at 0.055 a.u. Current density vectors are plotted onto the ACID isosurface to indicate diatropic ring currents. Magnetic field is orthogonal with respect to the C_5 ring plane.

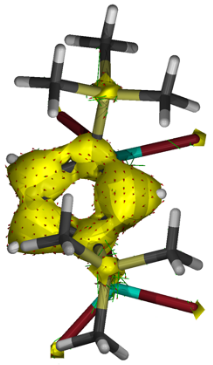

Fig. S61 | Total ACID isosurface of 3a at 0.05 a.u. Current density vectors are plotted onto the ACID isosurface to indicate diatropic ring currents. Magnetic field is orthogonal with respect to the C_5 ring plane.

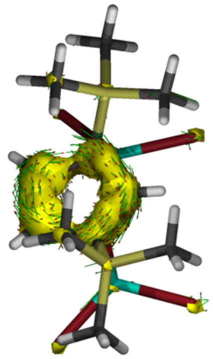

Fig. S62 | ACID isosurface of 3a at 0.05 a.u. Involving MOs with mainly π orbital contributions. Current density vectors are plotted onto the ACID isosurface to indicate diatropic ring currents. Magnetic field is orthogonal with respect to the C_5 ring plane.

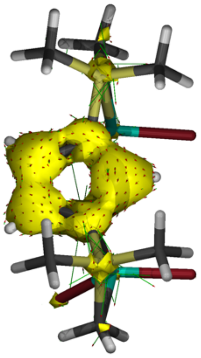

Fig. S63 | Total ACID isosurface of 3a$^+$ at 0.05 a.u. Current density vectors are plotted onto the ACID isosurface to indicate diatropic ring currents. Magnetic field is orthogonal with respect to the C_5 ring plane.

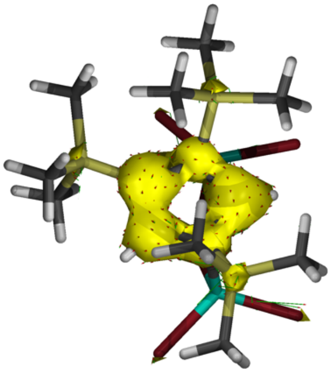

Fig. S64 | Total ACID isosurface of 3b at 0.05 a.u. Current density vectors are plotted onto the ACID isosurface to indicate diatropic ring currents. Magnetic field is orthogonal with respect to the C_5 ring plane.

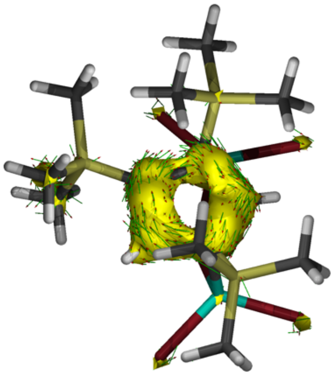

Fig. S65 | ACID isosurface of 3b at 0.05 a.u. Involving MOs with mainly π orbital contributions. Current density vectors are plotted onto the ACID isosurface to indicate diatropic ring currents. Magnetic field is orthogonal with respect to the C_5 ring plane.

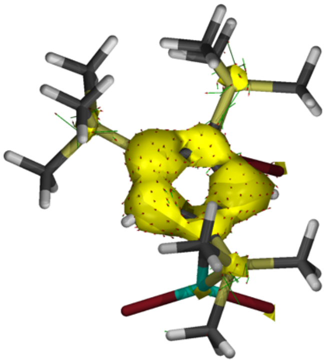

Fig. S66 | Total ACID isosurface of 3b$^+$ at 0.05 a.u. Current density vectors are plotted onto the ACID isosurface to indicate diatropic ring currents. Magnetic field is orthogonal with respect to the C_5 ring plane.

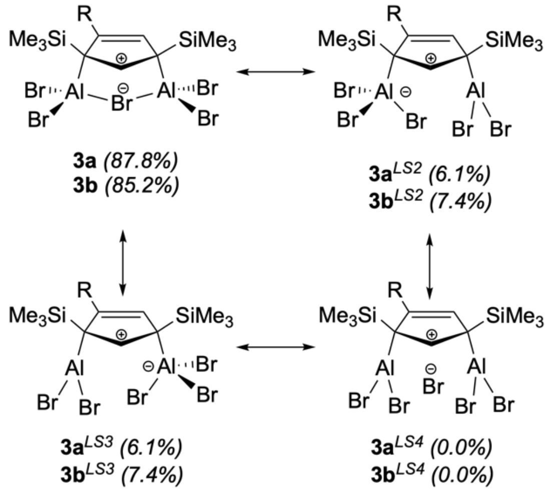

Fig. S67 | Results of natural resonance theory (NRT) analysis of 3a/b considering plausible Lewis structures **3a/b**, **3a/b**^{LS2}, **3a/b**^{LS3}, and **3a/b**^{LS4} (**3a** R = H; **3b** R = $SiMe_3$).

S4 Additional References

[1] A. W. Duff, P. B. Hitchcock, M. F. Lappert, R. G. Taylor, J. A. Segal, *J. Organomet. Chem.* **1985**, *293*, 271-283.

[2] C. P. Morley, P. Jutzi, C. Krüger, J. M. Wallis, *Organometallics* **1987**, *6*, 1084-1090.

[3] G. Sheldrick, *Acta Cryst.* **2015**, *A71*, 3-8.

[4] G. Sheldrick, *Acta Cryst.* **2008**, *A64*, 112-122.

[5] M. J. Frisch, G. W. Trucks, H. B. Schlegel, G. E. Scuseria, M. A. Robb, J. R. Cheeseman, G. Scalmani, V. Barone, B. Mennucci, G. A. Petersson, H. Nakatsuji, M. Caricato, X. Li, H. P. Hratchian, A. F. Izmaylov, J. Bloino, G. Zheng, J. L. Sonnenberg, M. Hada, M. Ehara, K. Toyota, R. Fukuda, J. Hasegawa, M. Ishida, T. Nakajima, Y. Honda, O. Kitao, H. Nakai, T. Vreven, J. A. Montgomery Jr., J. E. Peralta, F. Ogliaro, M. Bearpark, J. J. Heyd, E. Brothers, K. N. Kudin, V. N. Staroverov, R. Kobayashi, J. Normand, K. Raghavachari, A. Rendell, J. C. Burant, S. S. Iyengar, J. Tomasi, M. Cossi, N. Rega, J. M. Millam, M. Klene, J. E. Knox, J. B. Cross, V. Bakken, C. Adamo, J. Jaramillo, R. Gomperts, R. E. Stratmann, O. Yazyev, A. J. Austin, R. Cammi, C. Pomelli, J. W. Ochterski, R. L. Martin, K. Morokuma, V. G. Zakrzewski, G. A. Voth, P. Salvador, J. J. Dannenberg, S. Dapprich, A. D. Daniels, Ö. Farkas, J. B. Foresman, J. V. Ortiz, J. Cioslowski, D. J. Fox. *Gaussian 16*, Revision B.01, Gaussian, Inc.: Wallingford CT, **2016**.

[6] Y. Zhao, D. G. Truhlar, *J. Chem. Phys.* **2006**, *125*, 194101.

[7] Y. Zhao, D. G. Truhlar, *Acc. Chem. Res.* **2008**, *41*, 157-167.

[8] H. Braunschweig, J. O. C. Jimenez-Halla, K. Radacki, R. Shang, *Chem. Commun.* **2015**, *51*, 16569-16572.

[9] H. Braunschweig, J. O. C. Halla, K. Radacki, R. Shang, *Angew. Chem. Int. Ed.* **2016**, *55*, 12673-12677.

[10] C. Pranckevicius, J. O. C. Jiménez-Halla, M. Kirsch, I. Krummenacher, H. Braunschweig, *J. Am. Chem. Soc.* **2018**, *140*, 10524-10529.

[11] A. V. Marenich, C. J. Cramer, D. G. Truhlar, *J. Phys. Chem. B* **2009**, 113, 6378-6396.

[12] J. Baker, *J. Comput. Chem.* **1986**, *7*, 385-395.

[13] A. L. L. East, G. M. Berner, A. D. Morcom, L. Mihichuk, *J. Chem. Theory Comput.* **2008**, *4*, 1274-1282.

[14] A. Jayaraman, A. L. L. East, *J. Org. Chem.* **2012**, *77*, 351-356.

[15] E. D. Glendening, J. K. Badenhoop, A. E. Reed, J. E. Carpenter, J. A. Bohmann, C. M. Morales, P. Karafiloglou, C. R. Landis, F. Weinhold, *NBO 7.0.*, Theoretical Chemistry Institute, University of Wisconsin: Madison, WI, **2018**.

[16] G. Knizia, *J. Chem. Theory Comput.* **2013**, *9*, 4834-4843.

[17] R. D. Dennington II, T. A. Keith, J. M. Millam, *GaussView 6.0.16*, Gaussian, Inc.: Wallingford CT, **2016**.

[18] R. Herges, D. Geuenich, *J. Phys. Chem. A* **2001,** *105*, 3214-3220.

[19] D. Geuenich, K. Hess, F. Köhler, R. Herges, *Chem. Rev.* **2005**, *105*, 3758-3772.

[20] M. Giambiagi, M. S. de Giambiagi, K. C. Mundim, *Struct. Chem.* **1990**, *1*, 423.

[21] J. Kruszewski, T. M. Krygowski, *Tetrahedron Lett.* **1972**, *36*, 3839-3842.

[22] T. Lu, F. Chen, *J. Comput. Chem.* **2012**, *33*, 580-592.

[23] E. Matito, M. Duran, M. Solà, *J. Phys. Chem.* **2005**, *122*, 014109; Erratum, ibid **2006**, *125*, 055901.

[24] P. Bultinck, R. Ponec, S. Van Damme, *J. Phys. Org. Chem.* **2005**, *18*, 706-718.

[25] M. Giambiagi, M. S. de Giambiagi, C. D. dos Santos, A. P. Figueiredo, *Phys. Chem. Chem. Phys.* **2000**, *2*, 3381-3392.

[26] Matito, E. *ESI-3D: Electron Sharing Indices Program for 3D Molecular Space Partitioning*, Institute of Computational chemistry and Catalysis (IQCC), University of Girona, Catalonia, Spain, **2006** (http://iqc.udg.es/eduard/ESI).

[27] E. Matito, M. Solà, P. Salvador, M. Duran, *Faraday Discuss.* **2007**, *135*, 325-345.

[28] T. A. Keith, *AIMAll (Version 17.11.14)*, TK Gristmill Software, Overland Park KS, USA, **2017** (aim.tkgristmill.com).

[29] H. Fallah-Bagher-Shaidaei, C. S. Wannere, C. Corminboeuf, R. Puchta, P. v. R. Schleyer, *Org. Lett*. **2006**, *8*, 863-866.

S5 Cartesian coordinates of DFT optimized structures

**1^C**	C	-0.00036000	-1.19414900	0.00002700
	H	-0.00067400	-2.29000300	-0.00001800
	C	1.17307800	-0.35887400	0.00055400
	C	0.67244400	1.04508800	0.00111300
	C	-0.67183600	1.04550100	-0.00102800
	H	-1.32447200	1.91725300	-0.00197000
	C	-1.17322400	-0.35819100	-0.00054800
	H	1.32560600	1.91643200	0.00169700
	H	-1.85185300	-0.65552100	-0.83419700
	H	-1.83733100	-0.65348000	0.84652700
	H	1.83644200	-0.65436900	-0.84682100
	H	1.85167200	-0.65655900	0.83407200

**$C_5H_7^-$**	C	0.00001200	-1.23531000	-0.27139600
	H	0.00001500	-2.19834200	0.27870000
	C	1.20186100	-0.37914100	0.06787100
	C	0.66979400	1.02186100	-0.03394700
	C	-0.66976100	1.02185300	-0.03385900
	H	-1.29702800	1.92491400	-0.06336700
	C	-1.20187600	-0.37913300	0.06781800
	H	1.29698600	1.92498800	-0.06385600
	H	-2.10700300	-0.51523500	-0.58075600
	H	-1.66173300	-0.46091800	1.11530700
	H	2.10711100	-0.51547200	-0.58043200
	H	1.66146400	-0.46072200	1.11549100

**$C_5H_7^-$(fp)**	C	-0.04250700	-1.25203700	0.08666900
	H	-0.05915500	-2.00965600	-0.71568400
	C	1.21030600	-0.41674700	0.04678700
	C	0.70262900	0.99628600	-0.04233400
	C	-0.63978100	1.05299400	-0.05180800
	H	-1.22060900	1.98941200	-0.04367000
	C	-1.23228300	-0.36229500	0.04949600
	H	1.34768600	1.89116600	-0.03651600
	H	-1.94769000	-0.53539800	-0.79918900
	H	-1.94321100	-0.35119900	0.93944600
	H	1.90766200	-0.62893300	-0.80797900
	H	1.92513600	-0.46459700	0.93073600

**$C_5H_9^+$**	C	-0.00151400	1.24434900	0.00004500
	H	-0.00289500	2.34351400	0.00049300
	C	-1.18109400	0.43388000	-0.08888800
	C	-0.73847400	-1.00177800	0.19363800
	C	0.74090400	-1.00008500	-0.19361900
	H	1.34186300	-1.74269200	0.34254500
	C	1.17997300	0.43671400	0.08856800
	H	-0.87193500	-1.20311500	1.26792500
	H	2.09914800	0.85149400	-0.35390300
	H	1.36123000	0.58869800	1.19216700
	H	-1.36223200	0.58522800	-1.19233800
	H	-2.10129800	0.84633700	0.35397900
	H	-1.33799400	-1.74611900	-0.34157100
	H	0.87534100	-1.20182800	-1.26775900

**$C_5H_9^+$(fp)**	C	-0.00691400	1.23884000	-0.01136900
	H	-0.01167800	2.33853900	-0.02220500
	C	-1.19324800	0.44786600	0.00173100
	C	-0.77397200	-1.01779500	0.00219900
	C	0.77105700	-1.01467000	-0.00627900
	H	1.18620000	-1.55034000	0.85636700
	C	1.20538000	0.44880900	0.00595000
	H	-1.17952600	-1.53704000	0.87937300
	H	1.87342300	0.80002900	-0.81171500
	H	1.81168900	0.79527700	0.87496800
	H	-1.85636900	0.79734800	-0.82315000

	H	-1.82271900	0.80399800	0.85139500
	H	-1.18862900	-1.53998100	-0.86886800
	H	1.17379000	-1.52613300	-0.88956000
**C_5H_8**	C	0.00010100	-1.21247000	-0.13905200
	H	0.00019400	-2.12639000	0.47151100
	C	1.22358500	-0.31723400	0.10630100
	C	0.66903100	1.06695500	-0.04955000
	C	-0.66914000	1.06689000	-0.04938700
	H	-1.29615600	1.96005700	-0.10572400
	C	-1.22356500	-0.31740600	0.10626000
	H	1.29590000	1.96022000	-0.10605400
	H	-2.05760300	-0.53437100	-0.58063900
	H	-1.64271000	-0.45130900	1.12136500
	H	2.05793000	-0.53437000	-0.58015200
	H	1.64229800	-0.45085200	1.12163600
	H	0.00007900	-1.54339600	-1.18937700
**C_5H_8 (fp)**	C	-0.20462000	-1.17904400	-0.00780600
	H	-0.30151900	-1.86868000	0.84450600
	C	1.18062400	-0.51036700	0.00471000
	C	0.83455600	0.94611400	0.00074200
	C	-0.48619400	1.18451200	-0.00680600
	H	-0.95771400	2.16939600	-0.01060500
	C	-1.27916600	-0.14546300	0.00783800
	H	1.59720700	1.73038600	-0.00059600
	H	-1.96282700	-0.20598600	-0.85550100
	H	-1.93025400	-0.19823700	0.89690600
	H	1.79975800	-0.78689800	-0.86545200
	H	1.78212400	-0.78367800	0.88857600
	H	-0.29798500	-1.83082000	-0.88990700
**2a**	Br	-0.82032500	-1.56500900	1.67276600
	Al	0.11413200	-0.63947900	-0.21977900
	Br	1.48470200	-2.29706700	-1.03389800
	Si	-3.17276400	0.92592500	-0.33784000
	Si	2.69051100	1.68673000	0.37977700
	C	-1.30856100	0.97714100	-0.65661100
	C	-0.63396000	0.58539500	-1.86632200
	H	-1.11371600	0.16571400	-2.75250600
	C	0.75234800	0.83641800	-1.72580400
	H	1.51650600	0.57100600	-2.45619300
	C	1.00739200	1.32519900	-0.40550200
	C	-0.28310300	1.43960500	0.21932900
	H	-0.44731600	1.74757700	1.25439500
	C	2.82065400	0.63593900	1.92714200
	H	3.75572000	0.82964400	2.47339500
	H	2.80251700	-0.43934400	1.68857600
	H	1.99068300	0.82793400	2.62432400
	C	4.00798600	1.21738100	-0.86204800
	H	5.01687500	1.37275200	-0.45285900
	H	3.93541100	1.81307600	-1.78400800
	H	3.92856700	0.15598700	-1.14175500
	C	2.76388700	3.50713200	0.81674100
	H	3.71858200	3.76596100	1.29807400
	H	1.96036900	3.78458800	1.51494800
	H	2.65920800	4.14386000	-0.07350300
	C	-3.95039700	2.21087400	-1.46324800
	H	-5.04367500	2.23847400	-1.34248800
	H	-3.74408900	1.99711500	-2.52253900
	H	-3.56985300	3.22090200	-1.25293800
	C	-3.82343700	-0.77363400	-0.78340100
	H	-4.91787000	-0.81670100	-0.67941000
	H	-3.39983100	-1.55507100	-0.13582300
	H	-3.58988600	-1.03888400	-1.82575400
	C	-3.48277900	1.34310800	1.45804500
	H	-4.55796000	1.31639100	1.68828300
	H	-3.12295100	2.35189800	1.70949300
	H	-2.98284200	0.62848900	2.12798800

**2b**	Al	-0.42613200	0.07199700	0.65822700
	Br	0.21765800	-1.08302200	2.54130400
	Br	-1.69393100	1.81210700	1.50515700
	C	0.99226900	-0.76375500	-0.78556700
	C	-0.25762700	-1.44306200	-0.88943600
	H	-0.40619100	-2.50261900	-0.66559200
	C	-1.30714000	-0.52915700	-1.24525600
	C	-0.66235800	0.73270100	-1.40289000
	H	-1.18640800	1.66879800	-1.60291400
	C	0.72942400	0.63856300	-1.07412100
	Si	2.60998300	-1.70204400	-0.48100300
	C	3.46106100	-1.86569700	-2.14854300
	H	4.40448000	-2.42472400	-2.05428700
	H	3.69710700	-0.89621300	-2.60833400
	H	2.82458500	-2.41565900	-2.85748200
	C	3.74702700	-0.86768700	0.75256800
	H	4.63185100	-1.50268600	0.91306000
	H	3.25851500	-0.73619400	1.72915100
	H	4.11627300	0.11291500	0.42091900
	C	2.16587900	-3.41795900	0.12630400
	H	3.07836700	-3.99879000	0.32787700
	H	1.58788800	-3.98089800	-0.62171000
	H	1.58111200	-3.38862700	1.05691700
	Si	-3.15116000	-0.91739900	-1.40008000
	C	-3.70789600	-1.65855400	0.23051900
	H	-4.76572300	-1.95926100	0.20088000
	H	-3.59791600	-0.94024200	1.05797200
	H	-3.12369600	-2.55420900	0.49253500
	C	-4.05038200	0.68300900	-1.75676600
	H	-5.13571500	0.52315000	-1.83448600
	H	-3.71933900	1.13686200	-2.70262700
	H	-3.88300100	1.41812300	-0.95525700
	C	-3.37777100	-2.15932600	-2.78587200
	H	-4.43484000	-2.44053500	-2.90363800
	H	-2.81378600	-3.08391200	-2.59249400
	H	-3.03269900	-1.76046700	-3.75069900
	Si	1.85212100	2.16510900	-0.97535100
	C	0.85367800	3.59624300	-1.65743700
	H	1.43983700	4.52692300	-1.63909200
	H	-0.05017800	3.77284800	-1.05477000
	H	0.53780200	3.42871900	-2.69777300
	C	3.41080600	1.93152100	-1.99165800
	H	3.98681300	2.86903500	-2.01550400
	H	3.17980200	1.66321500	-3.03311400
	H	4.07845200	1.15638600	-1.59000800
	C	2.26363300	2.52060500	0.82133500
	H	2.97041600	3.36010700	0.90567600
	H	2.71150200	1.66418600	1.34493700
	H	1.35715000	2.80581400	1.37778900
**$AlBr_3$**	Al	0.00000000	0.00000000	0.00000000
	Br	0.00000000	2.23064400	0.00000000
	Br	-1.93179400	-1.11532200	0.00000000
	Br	1.93179400	-1.11532200	0.00000000
**3a**	Br	-2.53480700	-0.73729400	2.19023100
	Al	-1.93369600	-0.70808200	-0.00165900
	Br	-3.42261400	-1.51612300	-1.51660500
	Al	1.89733600	-0.74465000	0.02942800
	Br	-0.02762500	-2.22716600	-0.25384800
	Br	2.41688600	-0.70506800	2.24422400
	Br	3.40867900	-1.61929300	-1.42375900
	Si	-2.52617900	2.44235800	-0.17163800
	Si	2.64039600	2.35508800	-0.22460800
	C	-1.16003200	1.10792300	-0.54787300
	C	-0.67834700	0.90625300	-1.92059900
	H	-1.32978000	0.75976800	-2.78481700
	C	0.68754900	0.89170700	-1.93368600
	H	1.31840400	0.73195300	-2.81047300

	C	1.19935300	1.08919400	-0.57225900
	C	0.02913600	1.23113400	0.23212400
	H	0.04028700	1.40718400	1.31273000
	C	2.27301400	3.15181400	1.42585300
	H	3.06506700	3.87057100	1.68372200
	H	2.22585100	2.41527700	2.24095600
	H	1.32179300	3.70426500	1.40914000
	C	4.36294700	1.63375000	-0.23377600
	H	5.06962900	2.45244100	-0.02549700
	H	4.63857800	1.20112900	-1.20527500
	H	4.52807200	0.86471000	0.53483300
	C	2.48984700	3.60058800	-1.61432500
	H	3.20044500	4.42820600	-1.46903000
	H	1.48072600	4.03249500	-1.67186400
	H	2.71095600	3.14700200	-2.59117800
	C	-2.14277200	3.81867600	-1.38224000
	H	-2.81207000	4.67762100	-1.22398600
	H	-2.25845900	3.49700300	-2.42703700
	H	-1.10969800	4.17597900	-1.25769600
	C	-4.26829600	1.82329300	-0.42645100
	H	-4.96446700	2.65794800	-0.25059400
	H	-4.54833100	1.01816900	0.26919400
	H	-4.44454300	1.45604300	-1.44718100
	C	-2.28343700	3.04907700	1.57907100
	H	-3.06576400	3.78254600	1.82585800
	H	-1.31449900	3.55239800	1.71013200
	H	-2.35051700	2.24118200	2.32096600
**3a**[B]	Br	-2.33476800	-0.92426900	2.20503800
	Al	-1.83989200	-0.74596800	-0.01056100
	Br	-3.40539700	-1.47141500	-1.51321600
	Al	1.84001000	-0.74587800	-0.01145100
	Br	0.00003700	-2.26695400	-0.50691200
	Br	2.33853400	-0.92698800	2.20309700
	Br	3.40393500	-1.46804100	-1.51742900
	Si	-2.73838200	2.35949000	-0.05050000
	Si	2.73676200	2.36002400	-0.04797600
	C	-1.31451700	1.11868600	-0.50560000
	C	-0.70337900	1.37217500	-1.83044300
	H	-1.20524500	1.92996800	-2.63199000
	C	0.70351300	1.37247500	-1.82979100
	H	1.20592400	1.93044600	-2.63087100
	C	1.31354500	1.11901100	-0.50440400
	C	-0.00061600	1.82109500	-0.34174800
	H	-0.00080300	2.91112500	-0.17923400
	C	2.30930300	2.98349900	1.66558300
	H	3.09180800	3.66448200	2.03223300
	H	2.23281900	2.15396600	2.38371200
	H	1.36019700	3.53890700	1.69592500
	C	4.44372300	1.61101400	-0.05645600
	H	5.15589800	2.38094500	0.27877400
	H	4.75961700	1.27348100	-1.05265600
	H	4.54368200	0.75808000	0.63052300
	C	2.67172800	3.78696100	-1.27307400
	H	3.34166000	4.59414100	-0.94082800
	H	1.67001900	4.23415200	-1.37345400
	H	3.00832500	3.49367800	-2.27894600
	C	-2.67426400	3.78508700	-1.27716200
	H	-3.34484500	4.59213400	-0.94590700
	H	-3.01037300	3.49052600	-2.28282000
	H	-1.67282100	4.23284600	-1.37769000
	C	-4.44489300	1.60945100	-0.05739400
	H	-5.15732000	2.37937100	0.27732800
	H	-4.54395400	0.75730500	0.63070400
	H	-4.76118000	1.27048100	-1.05297500
	C	-2.31128800	2.98533400	1.66233400
	H	-3.09535300	3.66456300	2.02890900
	H	-1.36364800	3.54328700	1.69171600
	H	-2.23229600	2.15655300	2.38107000

**3a⁺**	Br	-2.09022100	-0.83369900	2.24932900
	Al	-2.13438700	-0.50908800	0.03450200
	Br	-2.36315800	-2.14889900	-1.45314800
	Al	2.13402100	-0.51037300	0.03378500
	Br	2.09252300	-0.83329400	2.24892700
	Br	2.35754700	-2.15183400	-1.45283200
	Si	-2.82126700	2.11600200	-0.30740400
	Si	2.82367900	2.11351800	-0.31022700
	C	-1.16800400	1.15366800	-0.63030400
	C	-0.68132500	0.95858800	-1.99985900
	H	-1.31851200	0.80555700	-2.87217600
	C	0.68108300	0.95820800	-2.00041800
	H	1.31746800	0.80479800	-2.87325300
	C	1.16898200	1.15314900	-0.63129000
	C	0.00088300	1.30062500	0.17343100
	H	0.00139200	1.47335200	1.25384100
	C	2.75585400	2.93539500	1.35075800
	H	3.71075400	3.43533300	1.56860000
	H	2.56374900	2.22641400	2.16775800
	H	1.97193100	3.70565800	1.37742100
	C	4.19918100	0.78057700	-0.34847200
	H	5.10169600	1.32497300	-0.02432200
	H	4.40907200	0.40716400	-1.36195600
	H	4.23900400	-0.08423000	0.34559400
	C	3.12857500	3.24867300	-1.74492900
	H	4.07119000	3.79754900	-1.60577300
	H	2.32308500	3.99016500	-1.83754900
	H	3.19699200	2.70599100	-2.69717000
	C	-3.12602300	3.25198700	-1.74147400
	H	-4.06781400	3.80202500	-1.60132800
	H	-3.19599100	2.70962900	-2.69379000
	H	-2.31966800	3.99247000	-1.83464100
	C	-4.19862900	0.78503700	-0.34432500
	H	-5.09998100	1.33058700	-0.01887600
	H	-4.23857400	-0.07983400	0.34964400
	H	-4.41037100	0.41208300	-1.35758700
	C	-2.75056100	2.93714700	1.35383500
	H	-3.70453900	3.43831800	1.57287800
	H	-1.96552200	3.70628200	1.38009900
	H	-2.55865700	2.22749500	2.17030900
**3b**	Br	-3.35961300	-1.02874200	-1.62420900
	Al	-1.55595100	-1.17085700	-0.24375400
	Br	-1.49144700	-3.03726000	1.05903600
	Al	2.15101500	-0.35840900	-0.60431600
	Br	3.29273700	0.80453300	-2.19244900
	Br	0.27576700	-1.29158100	-1.86735700
	Br	3.22038100	-2.09219900	0.40311000
	C	0.20376100	0.09182800	1.32927500
	H	0.36033000	-0.72159800	2.04573200
	Si	-2.41761400	0.66440500	2.25043200
	C	-1.07946500	0.50061400	0.84209200
	Si	-1.90047100	2.74633700	-1.16971100
	C	-0.81535200	1.63622300	-0.07555400
	Si	2.62978800	1.47156200	1.99216100
	C	0.54748200	1.83378100	-0.09210000
	H	1.06825100	2.59035900	-0.68801500
	C	1.24942300	0.87428000	0.76291700
	C	-1.79625700	-0.27643400	3.74377300
	H	-2.54416900	-0.22495300	4.54952400
	H	-0.86454100	0.15691600	4.13712900
	H	-1.61713700	-1.34063800	3.53526700
	C	-4.11701800	0.04698300	1.78449800
	H	-4.79998000	0.23440900	2.62757500
	H	-4.12860800	-1.03797300	1.59898700
	H	-4.54076400	0.53974300	0.89957900
	C	-2.42870900	2.49175700	2.66751200
	H	-3.09723200	2.68757600	3.51960000

	H	-2.75860600	3.12873800	1.83612800
	H	-1.42091700	2.82707700	2.95393700
	C	-3.74925700	2.65149900	-0.86885400
	H	-4.23662800	3.37314500	-1.54303700
	H	-4.05593400	2.92018900	0.15169400
	H	-4.16808300	1.66378400	-1.10392000
	C	-1.52049500	2.30125800	-2.95063800
	H	-2.00171400	3.00060100	-3.65079800
	H	-1.87229800	1.28944000	-3.20059000
	H	-0.43690400	2.32854400	-3.14330400
	C	-1.32710900	4.50421200	-0.82491500
	H	-1.89548600	5.23059200	-1.42524200
	H	-0.26397300	4.64526400	-1.06979400
	H	-1.45696500	4.77895900	0.23298000
	C	1.98745900	3.12560800	2.59419600
	H	2.66648400	3.56592600	3.33973300
	H	1.00133100	3.01519300	3.06926200
	H	1.87993900	3.85055700	1.77413300
	C	4.30004300	1.68706400	1.18682600
	H	5.00127800	2.08717700	1.93538700
	H	4.27683000	2.39467200	0.34612600
	H	4.72818600	0.74373600	0.81641800
	C	2.71564900	0.25830900	3.41048000
	H	3.51057400	0.55976300	4.10906700
	H	2.94565200	-0.76346700	3.07745500
	H	1.77663800	0.22628900	3.98189900
**3b**[B]	Br	-3.15391100	-1.38357400	-1.61004300
	Al	-1.39304800	-1.35442100	-0.13255800
	Br	-1.36078600	-3.05406700	1.38139300
	Al	2.17351900	-0.49563400	-0.42776600
	Br	3.43772200	0.32308000	-2.15533000
	Br	0.45477600	-1.68724700	-1.69013100
	Br	3.16916400	-1.92766500	1.03414800
	C	-0.01738500	1.19395500	1.12033900
	H	-0.13480500	1.94974800	1.91425200
	Si	-2.61524800	0.75974600	1.98863700
	C	-1.23951000	0.47646800	0.65772400
	Si	-2.12608800	2.76473200	-1.31482100
	C	-0.97831100	1.57494200	-0.32682400
	Si	2.59293100	2.08864100	1.49275200
	C	0.40290800	1.86399300	-0.38546300
	H	0.71424200	2.84206700	-0.77995100
	C	1.30830600	1.05186600	0.48236700
	C	-1.89850500	0.05297000	3.56947700
	H	-2.60902400	0.18416500	4.39961600
	H	-0.96418100	0.55429200	3.86400900
	H	-1.68424500	-1.02140400	3.48476400
	C	-4.23675500	-0.07941800	1.60193900
	H	-4.94194700	0.11643300	2.42432900
	H	-4.12026400	-1.17171900	1.53331900
	H	-4.70638100	0.25815400	0.66883800
	C	-2.84871400	2.60649000	2.29336200
	H	-3.47802800	2.73401800	3.18779600
	H	-3.34315600	3.15504300	1.48107100
	H	-1.90140200	3.12479900	2.51129100
	C	-3.94384900	2.36565900	-1.19281900
	H	-4.47397800	2.98159600	-1.93581200
	H	-4.40613100	2.57284100	-0.21948800
	H	-4.13743200	1.31450900	-1.44939100
	C	-1.52529500	2.43745000	-3.05757400
	H	-2.05211200	3.07060200	-3.78681700
	H	-1.70426900	1.38823900	-3.33714900
	H	-0.44783300	2.62780900	-3.17087500
	C	-1.75359400	4.52789700	-0.79534600
	H	-2.35579300	5.23453700	-1.38616100
	H	-0.69901600	4.79311600	-0.96168900
	H	-1.97965100	4.71638300	0.26398700
	C	2.04545600	3.88920900	1.43396900
	H	2.62326700	4.48764300	2.15415000

	H	0.98233400	4.03055100	1.68741300
	H	2.21125500	4.34012300	0.44360400
	C	4.34972500	1.94557900	0.88330900
	H	5.00131700	2.49708300	1.57872500
	H	4.49391700	2.36976400	-0.11928200
	H	4.71095600	0.90678300	0.85891200
	C	2.47254000	1.43593300	3.24455800
	H	3.22399700	1.91193500	3.89184800
	H	2.65447700	0.35114100	3.27291800
	H	1.48957100	1.62041800	3.70261300
**$3b^+$**	Br	-1.97054500	-0.66193000	-2.46932100
	Al	-1.53516400	-1.32236000	-0.38722800
	Br	-0.81159400	-3.40566400	0.01360000
	Al	2.39161000	0.17249000	-0.43933600
	Br	2.32073000	1.27475100	-2.37496200
	Br	3.07920300	-1.94848900	-0.25243500
	C	0.23935000	-0.23129300	1.35055300
	H	0.53815900	-1.17393200	1.82003000
	Si	-2.60080100	-0.50532700	2.04466200
	C	-1.09586900	0.11015900	0.97244000
	Si	-2.31650400	2.69805800	-0.34685800
	C	-1.04581700	1.47422900	0.39055900
	Si	2.68797300	1.29078700	2.05773800
	C	0.27098500	1.86622100	0.43692300
	H	0.65229700	2.82287700	0.06753800
	C	1.12863200	0.82580000	1.00485900
	C	-1.99917500	-1.76479700	3.27201300
	H	-2.84820300	-2.15089800	3.85530500
	H	-1.29687900	-1.31149300	3.98664500
	H	-1.50451500	-2.62919200	2.80957800
	C	-3.80459300	-1.30293100	0.80253800
	H	-4.62722700	-1.74190700	1.39050500
	H	-3.48471000	-2.18642200	0.21180400
	H	-4.26593700	-0.58169800	0.11244500
	C	-3.38445300	0.95672100	2.87742100
	H	-4.22006700	0.62383000	3.51054600
	H	-3.77338400	1.70783200	2.18002200
	H	-2.65256100	1.45265300	3.53117400
	C	-4.03067800	2.00750600	-0.67173300
	H	-4.62093200	2.82340800	-1.11766100
	H	-4.58802800	1.68795500	0.21951600
	H	-4.04214300	1.18890600	-1.40497100
	C	-1.58429900	3.30483700	-1.95849300
	H	-2.25859200	4.02967100	-2.43832400
	H	-1.41460200	2.48889800	-2.67548900
	H	-0.62177100	3.81564900	-1.80924500
	C	-2.42977800	4.13181600	0.85740300
	H	-3.08788200	4.91874600	0.45957700
	H	-1.44311900	4.59026700	1.02069200
	H	-2.82329100	3.84870400	1.84383900
	C	2.38782400	2.94841000	2.83570200
	H	3.25658400	3.25820300	3.43435400
	H	1.51739500	2.91504400	3.50557600
	H	2.20421100	3.72951000	2.08574200
	C	4.10763300	1.49626300	0.79329500
	H	5.01129900	1.63111100	1.41052700
	H	4.01461200	2.40407500	0.17850500
	H	4.44385400	0.67100200	0.13208000
	C	3.06379200	-0.08616600	3.24286500
	H	3.98080200	0.13319200	3.80867700
	H	3.21492700	-1.04930200	2.73571700
	H	2.25242400	-0.21167200	3.97410100
**TS1**	Br	3.73329900	0.76261900	-1.63495400
	Al	1.89888300	-0.15083200	-0.67219500
	Br	1.07901900	-1.96108400	-1.68833700
	Al	-2.34262000	-0.61925800	0.04589700
	Br	-2.02658800	-2.83990700	0.38979900

	Br	-1.96456400	0.28022200	-2.01619800
	Br	-4.26551300	0.12455700	1.02970900
	Si	3.23931200	-0.58941600	2.19482100
	Si	-0.77043900	3.24682300	0.26756000
	C	1.61185800	-0.02078000	1.34975300
	C	0.34401400	-0.54632900	1.69687200
	H	0.17267200	-1.53098600	2.13692500
	C	-0.67735700	0.37683000	1.36626700
	H	-1.66048200	0.38350500	1.86566400
	C	-0.08951900	1.54687800	0.78542200
	C	1.29658900	1.30292900	0.74530900
	H	2.05042700	2.06979400	0.53541700
	C	-0.03593700	3.64171900	-1.41033300
	H	-0.30624600	4.66065600	-1.72513700
	H	-0.39606500	2.95137100	-2.18658400
	H	1.06343100	3.58858800	-1.39906000
	C	-2.63207100	3.34413100	0.26895900
	H	-2.93273100	4.38465000	0.07205800
	H	-3.06638800	3.06135700	1.23870700
	H	-3.10191000	2.71712300	-0.50111600
	C	-0.08427900	4.44449000	1.54338100
	H	-0.39185300	5.47583200	1.31371100
	H	1.01515100	4.42975200	1.57246500
	H	-0.44446500	4.21496700	2.55687000
	C	2.77191100	-1.18090500	3.90678300
	H	3.66836100	-1.47897100	4.47072400
	H	2.10665700	-2.05555100	3.87115700
	H	2.26150800	-0.39666400	4.48376100
	C	4.01106200	-2.00230900	1.23498700
	H	4.89619400	-2.38991200	1.76141800
	H	4.34731300	-1.71170600	0.22743600
	H	3.30902000	-2.84241800	1.12082000
	C	4.36943800	0.89636000	2.27303000
	H	5.30966600	0.63916900	2.78277800
	H	3.90758300	1.72259200	2.83311600
	H	4.63441000	1.27198400	1.27414800
**INT**	Br	3.03778300	0.69276700	-2.17754900
	Al	1.79329400	-0.36431800	-0.59863600
	Br	2.58473100	-2.31245600	0.24743100
	Al	-1.70797300	-1.11785600	0.21727300
	Br	-1.33315500	-3.01748700	1.38885500
	Br	-0.32197300	-0.96623500	-1.75620300
	Br	-3.84338200	-0.84434400	-0.50042100
	Si	2.55087700	1.74125700	1.70669200
	Si	-2.53672800	2.90706000	-0.03433700
	C	1.04389100	0.91611700	0.78456900
	C	0.15404200	0.13061700	1.59397300
	H	0.45463000	-0.68600800	2.25620400
	C	-1.17177500	0.57576800	1.39237900
	H	-1.99571200	0.33104400	2.08164500
	C	-1.15150100	1.73268400	0.50052400
	C	0.16799000	1.89595800	0.13801200
	H	0.53912600	2.65278000	-0.55916900
	C	-2.92113000	2.65689800	-1.85031100
	H	-3.65836300	3.39338700	-2.20357100
	H	-3.32087600	1.65631800	-2.06458500
	H	-2.01215800	2.78688700	-2.45738300
	C	-4.02851400	2.67645400	1.07338200
	H	-4.81985800	3.39499500	0.81282100
	H	-3.76985900	2.85122600	2.12883600
	H	-4.46474400	1.67072400	0.99566100
	C	-1.84622100	4.63921000	0.20209700
	H	-2.58968400	5.40410300	-0.06829600
	H	-0.96003100	4.81578400	-0.42585300
	H	-1.55148800	4.82195800	1.24629400
	C	1.88675800	2.20137800	3.39527900
	H	2.61223500	2.81242100	3.95283000
	H	1.66396300	1.31569200	4.00774500
	H	0.95979500	2.78788000	3.30622100

	C	4.02327100	0.60483500	1.86405800
	H	4.80911500	1.12354500	2.43459700
	H	4.46015900	0.33064000	0.89181100
	H	3.79078400	-0.32766900	2.39732500
	C	2.99436200	3.28290900	0.74999100
	H	3.88200100	3.75225800	1.20030800
	H	2.18286600	4.02481600	0.77575000
	H	3.23365700	3.07350200	-0.30215100
**TS2**	Br	3.18759200	0.00491100	-2.18846400
	Al	2.02056300	-0.57445400	-0.32592600
	Br	3.05247800	-1.93628900	1.17085400
	Al	-1.78577900	-0.86381000	0.14563300
	Br	-2.35447300	-2.38203100	1.75365500
	Br	0.06148400	-1.81485500	-1.12355200
	Br	-3.40812100	-0.76830300	-1.46274300
	Si	2.37928100	2.32789300	0.95952500
	Si	-2.72981700	2.59660000	-0.03215300
	C	1.03325200	0.97110100	0.53114500
	C	0.33402800	0.43225300	1.67820600
	H	0.80342500	-0.09668500	2.51114000
	C	-1.02794500	0.68777400	1.55192600
	H	-1.76638200	0.51888800	2.34107600
	C	-1.27247200	1.41310800	0.29860500
	C	-0.01487900	1.53716500	-0.29902600
	H	0.17296100	2.02768200	-1.25838200
	C	-2.91835100	2.91831000	-1.86159500
	H	-3.70131400	3.67117500	-2.03608500
	H	-3.18886300	2.01736300	-2.42722200
	H	-1.98833300	3.31987200	-2.29086200
	C	-4.28675200	1.99486400	0.81482800
	H	-5.04916800	2.78806800	0.78838700
	H	-4.10493500	1.77263300	1.87769100
	H	-4.72831000	1.10168700	0.35383300
	C	-2.19061600	4.18848000	0.81339900
	H	-2.95387900	4.97387100	0.70374600
	H	-1.25495500	4.57897800	0.38469200
	H	-2.02744200	4.04256200	1.89188700
	C	1.66040900	3.28276500	2.39868800
	H	2.27699600	4.16453600	2.62921900
	H	1.60617200	2.67169200	3.31108900
	H	0.64438200	3.63969500	2.17524900
	C	4.01388600	1.56746000	1.44174600
	H	4.70377000	2.37732400	1.72552300
	H	4.49346600	1.00789900	0.62466800
	H	3.92931400	0.89093000	2.30384700
	C	2.55532100	3.43996500	-0.53005400
	H	3.37221200	4.15693300	-0.35818900
	H	1.64131400	4.02413900	-0.71126700
	H	2.79602400	2.88572400	-1.44789100
**3b'**	Br	1.46636900	-3.06715700	0.99508400
	Al	1.52493300	-1.18492100	-0.28683600
	Br	3.31114200	-0.97788200	-1.67780500
	Al	-2.18276600	-0.33306700	-0.58775300
	Br	-3.27779600	-2.06158200	0.39927200
	Br	-0.33001600	-1.28150500	-1.88150800
	Br	-3.32269800	0.86928800	-2.14757800
	C	-0.52260200	1.81743300	-0.07955200
	H	-1.04089700	2.58089300	-0.66916500
	Si	1.92147900	2.74194100	-1.14653900
	C	0.83939200	1.61335300	-0.06863400
	Si	2.47158800	0.56527800	2.22718900
	C	1.10066200	0.47330500	0.84313600
	Si	-2.54311400	1.48855700	2.05110800
	C	-0.18162800	0.06491500	1.32860600
	H	-0.34142900	-0.75231600	2.03949900
	C	-1.22681100	0.85667300	0.77188200
	C	1.42065600	2.43911900	-2.92761600

	H	1.90730700	3.15405900	-3.60805600
	H	0.33283400	2.53586300	-3.06477200
	H	1.70409800	1.42786000	-3.25531000
	C	3.78143300	2.53550900	-0.95489600
	H	4.19989200	1.91933200	-1.76205200
	H	4.09489900	2.07434200	-0.00913600
	H	4.26912600	3.52098300	-1.00583500
	C	1.44557800	4.49368200	-0.65133300
	H	1.98818700	5.23703600	-1.25490200
	H	1.67416100	4.69889100	0.40557200
	H	0.37101800	4.68144800	-0.79406800
	C	4.11337900	-0.22312900	1.80515100
	H	4.02456100	-1.30911800	1.64990800
	H	4.78917000	-0.07885000	2.66269800
	H	4.60973300	0.19508500	0.91982200
	C	1.77146800	-0.27746400	3.74496100
	H	2.51413400	-0.26029200	4.55690800
	H	1.51701200	-1.33154400	3.56179000
	H	0.86995200	0.23357700	4.11533300
	C	2.65805400	2.39575500	2.58747500
	H	1.68300300	2.85192800	2.81399100
	H	3.10085900	2.96446500	1.75852500
	H	3.30492000	2.54242500	3.46594600
	C	-1.76343300	3.05333900	2.72657100
	H	-2.39360000	3.51074100	3.50396300
	H	-1.59990000	3.80839700	1.94366100
	H	-0.78606600	2.83188800	3.18200100
	C	-2.71594200	0.22701900	3.41815500
	H	-3.46626800	0.57433500	4.14442000
	H	-1.77563300	0.08217900	3.96949300
	H	-3.04774500	-0.75373500	3.05048600
	C	-4.19202600	1.85964500	1.26011200
	H	-4.86356600	2.29132600	2.01806100
	H	-4.68810500	0.96127200	0.86303700
	H	-4.11328300	2.58492700	0.43784800

**TS$_{3b-3b'}$**	Br	1.18405900	-2.79050900	1.25025200
	Al	1.26090700	-1.12655400	-0.33232600
	Br	2.99616700	-1.47374000	-1.76811700
	Al	-2.34156200	-0.41717500	-0.24775300
	Br	-2.99049900	-1.77825300	1.44403100
	Br	-0.72706800	-1.62479500	-1.66608700
	Br	-3.95419200	0.26086200	-1.70330500
	C	-0.19164600	1.44853200	-0.72246800
	H	-0.55419000	1.85707900	-1.67167300
	Si	2.41854800	2.19193500	-1.46930800
	C	1.15165200	1.22983400	-0.41097600
	Si	2.59270800	0.66384900	2.25962800
	C	1.17489100	0.69141500	0.97487300
	Si	-2.14324600	2.53236700	1.02494200
	C	-0.16595100	0.54574800	1.36632200
	H	-0.48913900	0.12558000	2.32388700
	C	-1.07298700	1.04530300	0.35437800
	C	2.01519500	1.87731300	-3.27071900
	H	2.66997900	2.47840700	-3.91934700
	H	0.98003900	2.15652100	-3.51655300
	H	2.15173500	0.82337400	-3.55024200
	C	4.23881400	1.90462300	-1.13358400
	H	4.79919100	2.61940800	-1.75681700
	H	4.58586700	0.89887500	-1.40165700
	H	4.53139100	2.10238500	-0.09314800
	C	2.05243300	3.99847600	-1.08014000
	H	2.70699400	4.66919900	-1.65759100
	H	2.20125000	4.23737900	-0.01625800
	H	1.01318600	4.25959900	-1.33127300
	C	4.07989400	-0.34606100	1.74354900
	H	4.88627000	-0.19522300	2.47784500
	H	4.48134900	-0.06924200	0.75965900
	H	3.85836100	-1.42275100	1.72046900

	C	1.86957700	0.00772600	3.85453600
	H	2.64781300	-0.04556900	4.63031800
	H	1.45712000	-1.00431200	3.73524900
	H	1.07165800	0.65849500	4.24244400
	C	3.06969800	2.46511900	2.50672900
	H	3.85437300	2.54990200	3.27401200
	H	2.20971600	3.05798100	2.85232600
	H	3.45570300	2.94479100	1.59642800
	C	-0.97469700	3.50739200	2.11761800
	H	-1.43331000	4.45281700	2.44430200
	H	-0.05148100	3.76048900	1.57366200
	H	-0.68894000	2.94952800	3.02187900
	C	-3.59514100	1.89313800	2.00842200
	H	-4.12780800	2.73903200	2.46904200
	H	-3.28507400	1.21882700	2.81983900
	H	-4.32520500	1.35091700	1.38863200
	C	-2.67385400	3.59414800	-0.41569900
	H	-3.31931200	4.40663600	-0.04889200
	H	-3.24360200	3.03964900	-1.17319400
	H	-1.81119100	4.06361100	-0.91008700
**C_6H_6**	C	-1.22757500	-0.66246100	0.00001000
	C	-0.03996200	-1.39432100	-0.00006800
	C	1.18749000	-0.73186100	0.00001700
	C	1.22752400	0.66255000	-0.00003000
	C	0.04006600	1.39431500	-0.00000100
	C	-1.18754400	0.73178000	0.00001600
	H	-2.18887400	-1.18137800	0.00000400
	H	-0.07143400	-2.48628500	0.00021100
	H	2.11756100	-1.30484300	0.00001200
	H	2.18894900	1.18122300	0.00010700
	H	0.07128400	2.48628000	0.00001500
	H	-2.11748200	1.30499100	-0.00001300
**NC_5H_5**	C	-1.13928600	-0.72300900	-0.00020600
	C	-1.19705300	0.67129200	-0.00011100
	C	0.00001300	1.38326900	0.00008300
	C	1.19706300	0.67127300	0.00022000
	C	1.13927300	-0.72302800	0.00010800
	N	-0.00001200	-1.41474700	-0.00010200
	H	0.00002100	2.47610400	0.00015300
	H	-2.06520100	-1.31093000	-0.00036600
	H	-2.16072500	1.18514000	-0.00021700
	H	2.16074900	1.18509600	0.00038800
	H	2.06517900	-1.31096200	0.00019600
**BC_5H_5**	C	-1.35647800	-0.83189600	-0.00024100
	C	-1.22879700	0.57612600	-0.00013400
	C	-0.00000900	1.24966200	0.00008200
	C	1.22878500	0.57613700	0.00020700
	C	1.35649100	-0.83188400	0.00013200
	H	-0.00001200	2.34114000	0.00014500
	H	-2.33567000	-1.30316100	-0.00038800
	H	-2.14918300	1.17232800	-0.00021200
	H	2.14916400	1.17235000	0.00039200
	H	2.33569600	-1.30312200	0.00025600
	B	0.00001000	-1.30168100	-0.00009500
**$B_3N_3H_6$**	B	1.43583600	0.18796800	0.00000700
	H	2.62781700	0.34391600	0.00002600
	B	-0.55513000	-1.33742500	-0.00000500
	H	-1.01605700	-2.44769200	-0.00002700
	B	-0.88069800	1.14945200	0.00000100
	H	-1.61173300	2.10376800	0.00000100

	H	1.46952500	-1.91809200	0.00000200
	H	0.92635300	2.23168400	-0.00002500
	H	-2.39590900	-0.31355100	0.00002600
	N	0.53864600	1.29769600	-0.00000600
	N	0.85453700	-1.11532100	-0.00000100
	N	-1.39318700	-0.18237700	0.00000500

**C_6H_{12}**	C	-1.25572200	-0.73049900	-0.22821500
	C	0.00489700	-1.45274800	0.22812700
	C	1.26056200	-0.72207300	-0.22814300
	C	1.25573300	0.73054600	0.22812800
	C	-0.00494300	1.45273600	-0.22815900
	C	-1.26052600	0.72203800	0.22826000
	H	2.16381200	-1.23921700	0.13262300
	H	0.00520800	-1.51736300	1.33171700
	H	0.00864600	-2.49362000	-0.13250200
	H	-1.31142700	-0.76298800	-1.33181000
	H	-2.15527000	-1.25424300	0.13234400
	H	1.31151800	0.76319600	1.33170600
	H	2.15523000	1.25425300	-0.13256500
	H	-0.00869000	2.49362900	0.13242400
	H	-0.00538900	1.51730000	-1.33175400
	H	-1.31661200	0.75398800	1.33183700
	H	-2.16385200	1.23923100	-0.13231300
	H	1.31681700	-0.75416300	-1.33169600
